# Comparison of Microarray Platforms for Measuring Differential MicroRNA Expression in Paired Normal/Cancer Colon Tissues

**DOI:** 10.1371/journal.pone.0045105

**Published:** 2012-09-13

**Authors:** Maurizio Callari, Matteo Dugo, Valeria Musella, Edoardo Marchesi, Giovanna Chiorino, Maurizia Mello Grand, Marco Alessandro Pierotti, Maria Grazia Daidone, Silvana Canevari, Loris De Cecco

**Affiliations:** 1 Functional Genomics Core Facility, Fondazione IRCCS Istituto Nazionale dei Tumori, Milan, Italy; 2 Unit of Biomarkers, Fondazione IRCCS Istituto Nazionale dei Tumori, Milan, Italy; 3 Cancer Genomic Laboratory, Edo ed Elvo Tempia Foundation, Biella, Italy; 4 Unit of Molecular Therapies, Department of Experimental Oncology and Molecular Medicine, Fondazione IRCCS Istituto Nazionale dei Tumori, Milan, Italy; 5 Scientific Directorate, Fondazione IRCCS Istituto Nazionale dei Tumori, Milan, Italy; Mount Sinai School of Medicine, United States of America

## Abstract

**Background:**

Microarray technology applied to microRNA (miRNA) profiling is a promising tool in many research fields; nevertheless, independent studies characterizing the same pathology have often reported poorly overlapping results. miRNA analysis methods have only recently been systematically compared but only in few cases using clinical samples.

**Methodology/Principal Findings:**

We investigated the inter-platform reproducibility of four miRNA microarray platforms (Agilent, Exiqon, Illumina, and Miltenyi), comparing nine paired tumor/normal colon tissues. The most concordant and selected discordant miRNAs were further studied by quantitative RT-PCR. Globally, a poor overlap among differentially expressed miRNAs identified by each platform was found. Nevertheless, for eight miRNAs high agreement in differential expression among the four platforms and comparability to qRT-PCR was observed. Furthermore, most of the miRNA sets identified by each platform are coherently enriched in data from the other platforms and the great majority of colon cancer associated miRNA sets derived from the literature were validated in our data, independently from the platform. Computational integration of miRNA and gene expression profiles suggested that anti-correlated predicted target genes of differentially expressed miRNAs are commonly enriched in cancer-related pathways and in genes involved in glycolysis and nutrient transport.

**Conclusions:**

Technical and analytical challenges in measuring miRNAs still remain and further research is required in order to increase consistency between different microarray-based methodologies. However, a better inter-platform agreement was found by looking at miRNA sets instead of single miRNAs and through a miRNAs – gene expression integration approach.

## Introduction

microRNAs (miRNAs) are small non-coding RNA molecules of 18–24 nucleotides in length that are widely conserved in all eukaryotic organisms and serve as regulators of gene expression. miRNAs are involved in all major cellular processes and are implicated in a large number of human diseases including cancer [1]–[3].

Over the past decade, DNA microarray technology has become an increasingly cost-effective methodology that is able to quickly generate high-throughput data, paving the way to genome-wide (GW) analysis of gene-expression, genomic copy number variations, SNPs, and epigenetic alterations. Microarray-based techniques have been extensively used in several areas of research and molecular assays using patterns of gene expression and predetermined mathematical algorithms, such as Mammaprint®) [4], are currently under validation by prospective multicentric clinical studies in breast cancer.

More recently, microarray technology has been applied to miRNA profiling and is becoming a promising technique in many research fields, such as translational research in oncology, and can provide useful information on the role of miRNAs in both tumorigenesis and progression of cancer [2]. Nevertheless, independent studies characterizing the same pathology have often poorly overlapping results. This could be due to small sample size, high tumor variability and heterogeneity but also to technical reasons. A major advantage of the microarray approach consists on the high-throughput simultaneous screening of up to thousands molecules in a single assay, but this requires hybridization conditions to be the same for all probes on the array. This is not trivial for miRNA microarrays because the GC content of miRNAs is highly variable and the options for probe design are more limited than for mRNA due to their short length. For a complete review of general concepts and special challenges that are relevant to miRNA profiling refers to Pritchard et al [5]. A multitude of platforms for miRNA profiling are commercially available, and each manufacturer has developed its own technical procedures to maximize sensitivity and specificity in measuring miRNA expression levels. As a result, probe signals are expected to largely differ among platforms, and a direct comparison is not possible. In spite of this, the general patterns of differentially expressed (DE) miRNAs should be coherently detected by all platforms. Only recently the comparison of intra- and inter-platform reproducibility of miRNA microarrays has been analyzed in more than three different platforms (see Table S1 for details) [6]–[11]. Taken together, these studies provide evidence that miRNA microarray platforms show excellent intra-platform reproducibility, but limited inter-platform concordance. Indeed, comparing miRNAs identified as DE within each platform, a significant variation in the total number as well as in the fold-change of miRNAs has been noted. Three of these studies [6]; [8]; [9] based their conclusions on the comparison of tissues or pools of tissues of completely different origin. Sah et al. analyzed the expression of seven synthetic miRNAs spiked in known concentration into a RNA from placental tissue and hybridized on five platforms [11]. To be nearer to a miRNA microarray application in cancer research, Git et al. analyzed a pool of normal breast tissues and two breast cancer cell lines [7] and Dreher et al. compared untrasfected and HPV-transfected human keratinocytes [10]. Even if, the former four comparisons represent a useful system to address technical issues, and the later two studies are undoubtedly more realistic, the issue of concordance of different platforms, when clinically specimens are used, has not been yet addressed.

In the present study, we compared the miRNA expression profiles of nine colorectal cancer and normal colon mucosa samples from the same patients using four different commercial platforms (Agilent, Exiqon, Illumina and Miltenyi). The expression of the most concordant and selected discordant miRNAs among platforms was then evaluated with quantitative real time PCR (qRT-PCR). Finally, integrative analyses of miRNAs in the context of gene expression and literature data were performed as a proof of principle of the validity of microarray miRNA analysis in gaining insight into the biological role of these miRNAs.

## Results

### Experimental Setting

To highlight the influence of the sample origin and the study design on the obtained results, we made a computational comparison of expression data from four microarray studies. We selected the data obtained on a common miRNA platform, i.e Agilent, from the two miRNA platform comparison studies (details in Table S1) whose expression data on human samples are publicly available (GSE13860 [6] and E-MTAB-96 [7]) and from two studies chosen as examples of experimental applications in a clinical setting, i.e. profiles associated with tumorigenesis of prostate [12] and gastric [13] cancer (GSE21036 and GSE28700, respectively; details in Fig.S1 legend). As shown in Figure S1, the number of DE miRNAs and the associated fold changes are considerably higher in the cross-platform analysis than in profiles looking at tumorigenesis. Imposing a uniform and arbitrary threshold (|log_2_ fold change|>1), 88.5% (GSE13860) and 25.9% (E-MTAB-96) of miRNAs present in the arrays were differentially expressed in the cross-platform datasets; on the other hand, only 6.9% (GSE21036) and 6.7% (GSE28700) of miRNAs were identified as DE at same threshold in the clinical datasets.

With these premises, we decided to evaluate the inter-platforms reproducibility in a clinical setting by assessing the tumor and the normal counterpart miRNA profiles in samples collected from nine patients who underwent surgical resection for colon cancer (see Table S2 for clinical and pathological characteristics). RNA aliquots from these samples were hybridized on four microarray platforms: Agilent SurePrint G3 human miRNA Microarray, Exiqon miRCURY LNA microRNA Array, Illumina Human_v2 microRNA expression Beadchips, and Miltenyi miRXplore Microarray. Main features of the four platforms are described in Table 1.

**Table 1:** Platform description.

	Agilent	Exiqon	Illumina	Miltenyi
Array version	Human miRNA V3	miRCURY LNA microRNA Array	Human miRNA_V2	miRXplore microarray V5
Array per slide	8	1	12	1
Channels	Single	Single	Single	Dual
Input total RNA	100 ng	300 ng	600 ng	1200 ng
Labeling	Cy3	Hy3	Cy3	Hy3/Hy5
Labeling process	Alkaline phosphatase and 3′ ligation	Alkaline phosphatase and3′ ligation	Polyadenylation, RT, MSO§pool annealing, PCR	Alkaline phosphatase and3′ ligation
miRBase version	miRBase V12.0	miRBase V14.0	miRBase V12.0	miRBase V14.0
N° hsa-miR	866	891	858	911
N° probes/miR	2	1	1	1
N° replicates/probe	4–8	4	370 (average)	4

§ MSO = miRNA specific oligo.

It should be noted that the platform from Illumina was withdrawn since March 2010; however, we decided to include it in our comparison due to its extensive use in laboratories worldwide, including those in our Institute. Accordingly, the issues addressed in the present investigation can be of interest to users of the Illumina platform to better interpret their results and to enable a more rationale switch to a different platform.

The Agilent, Exiqon, and Illumina arrays were carried out in one-color. Miltenyi was hybridized in two colors: tissue samples were labeled with Hy5, and a synthetic reference purchased by Miltenyi with Hy3. Since the synthetic reference was designed on miRBase 9.2 and covered only a portion of the miRNAs present on the arrays designed on miRBase 14.0, only the Hy5 data were considered and used for normalization in order to enable a more direct comparison with the other three platforms.

The Agilent, Exiqon, and Illumina platforms contained probes designed either on viral miRNA sequences or on putative miRNAs not yet annotated in miRBase, derived from literature and Next-Generation Sequencing studies. Since these sequences are present only in one platform, they were excluded from our analyses.

miRBase database is the primary repository for all miRNA sequences and annotations used by all manufacturers for the design of the probes. However, the frequent update of miRBase results in annotation problems. To avoid possible bias, we selected arrays designed on close miRBase versions and the probes of the four tested platforms were designed on either v12.0 or v14.0 miRBase. We verified that the names and sequences of miRNAs present in v12.0 did not change in the newer miRBase version, while a set of new miRNAs was added. The difference in the total number of miRBase annotated miRNAs in the four platforms was relatively small (6%).

### Evaluation of Data Distribution and Detection Rate

**Figure 1:**
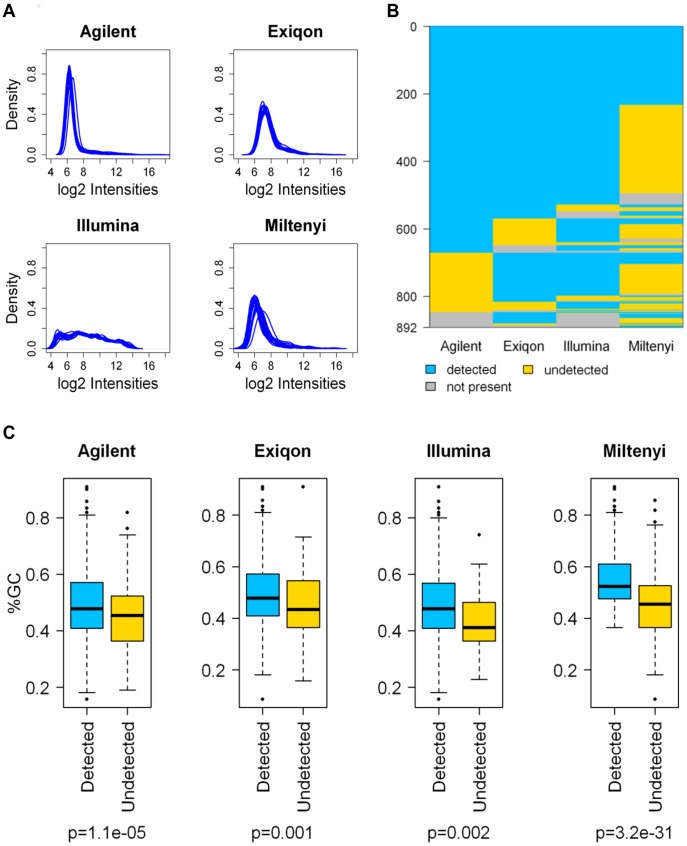
Comparison of microarray platform performance. (A) Global non-normalized intensity distribution. (B) Graphical representation of miRNA detection; blue = detected, yellow = undetected, gray = not present. (C) Box-plot of the percentage of GC content in mature miRNA sequences; blue = detected, yellow = undetected. *P*-values were calculated by Student’s t test.

Non-normalized signal intensities showed a platform dependent distribution reflecting the unique methods developed by manufacturers for labeling, hybridization stringency and data acquisition (Fig. 1A). For all platforms, the signals covered most of the dynamic range available for 16-bit scanners; Agilent, Exiqon, and Miltenyi signal distributions tended to have positive skewness (a right side long tail) and differed from Illumina distributions where many more probes showed intermediate to high expression levels.

For the Agilent and Illumina platforms, we followed the detection call criteria recommended by the manufacturers. Illumina’s software provides a detection *P*-value that estimates to what extent a signal is greater than the noise represented by negative controls; similarly, Agilent’s software provides a flag (gIsPosAndSignif) that estimates if the feature signal is positive and significant compared to the background. In contrast, for the Exiqon and Miltenyi platforms a detection call criteria was not defined; for these platforms, we established a threshold percentage of pixels for every spot in the array whose intensity was lower than background. Taking into account these filtering procedures, 675 (78% of miRBase annotated miRNAs present on the array), 775 (87%), 808 (94%), and 376 (41%) unique miRNAs were detectable in at least one of the samples in the Agilent, Exiqon, Illumina, and Miltenyi platforms, respectively (Fig. 1B). There were 233 miRNAs that were shared by all platforms, being strongly limited by the low detection rate in the Miltenyi platform.

In order to estimate to what extent the GC content impacted the detection call of each platform, we calculated the GC percentage of the miRNAs assayed and compared, for each platform, the GC content between detected and undetected miRNAs. Despite each manufacturer has adjusted probe design and hybridization procedures to overcome discrepancies in the thermodynamic stability of probe/target recognition, the GC content was significantly higher in the detected than in the undetected miRNAs in all platforms, and this difference was particularly evident for the Miltenyi platform (Fig. 1C).

### Normalization and Class Comparison Results

Several normalization and data processing procedures are available, most translated by gene-expression studies and with little consensus among laboratories. Considering the unique characteristics of each platform, it is unlikely that the same normalization procedure could perform equally in all platforms to correct systematic differences.

In order to choose the best normalization for each platform, we evaluated the ability of the four different methods (loess, quantile, rank invariant, and Robust Spline Normalization) to reduce the intra-class variability in normal and tumor samples through the use of Relative Log Expression (RLE) (see Fig. S2). Moreover, we expected that the best normalization method should increase the fold changes and the number of differentially expressed miRNAs between tumor and normal tissue. According to these criteria, we chose RSN for Illumina and Agilent, loess for Exiqon and quantile for Miltenyi.

In Figure S3 the tumor/normal class comparisons in the 4 platforms, expressed as histograms of log *P*-value and FDR, are reported. The comparison identified, at a threshold *P*<0.005, 29 miRNAs that were modulated on Agilent, 4 on Exiqon, 42 on Illumina, and 3 on the Miltenyi platform, corresponding to 4.3%, 0.5%, 5.2%, and 0.8% of miRNAs detected, respectively.

### Inter-platform Agreement of Class Comparison Results

**Figure 2:**
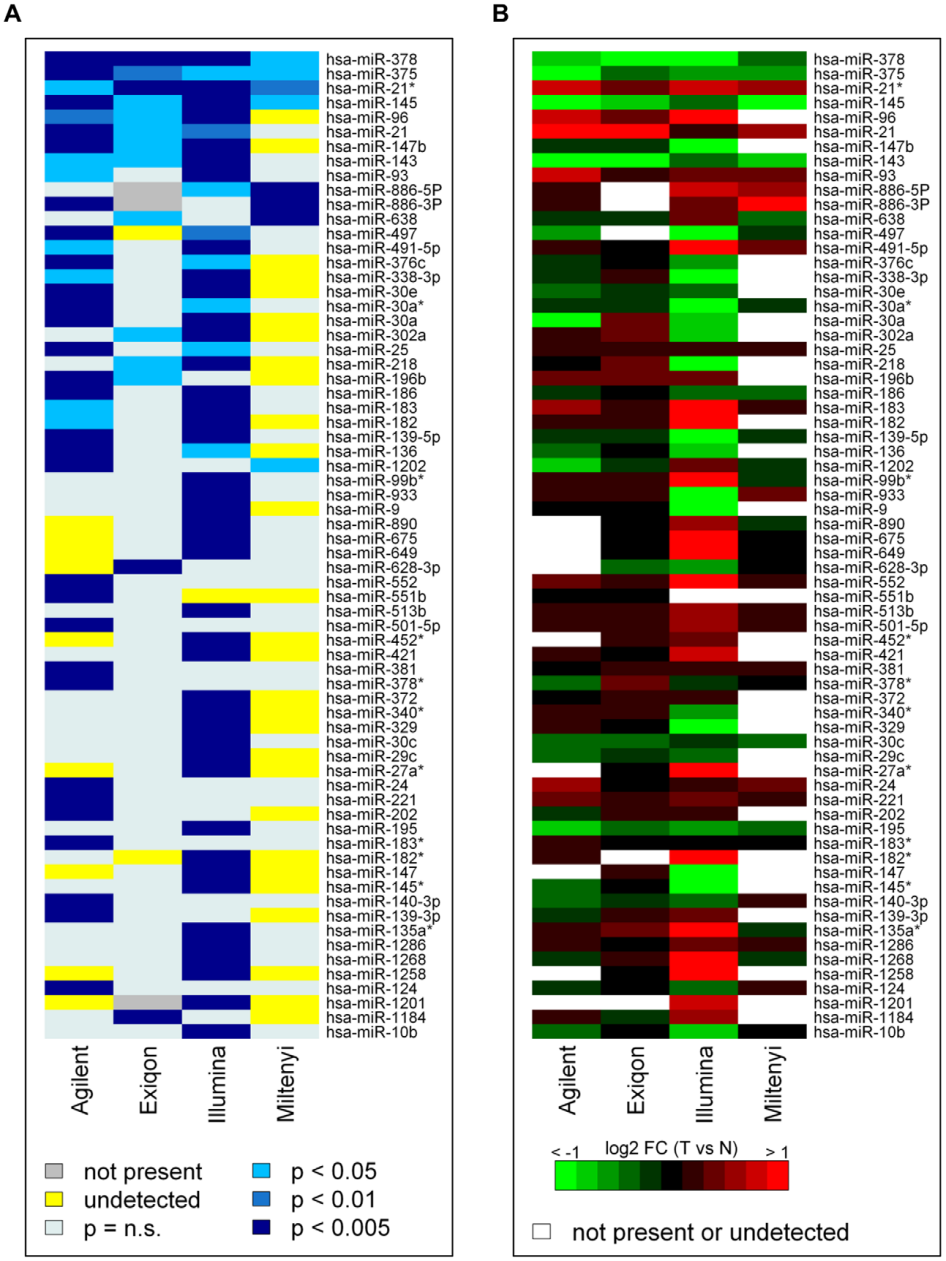
Cross-platform comparison of the consensus list of DE miRNAs at *P*<0.005 in at least one platform. (A) *P*-values of the tumor/normal class comparison visualized in a blue-white heat map; see scale in the figure. (B) Log_2_ fold changes in the tumor/normal class comparison visualized in a red-green heat map; red = up-regulated; green = down-regulated in tumors.

To assess inter-platform concordance, we examined the miRNAs that were DE at *P*<0.005 in at least one platform; by combining these miRNAs, a consensus list of 68 miRNAs was generated. To highlight concordance among the four platforms, the *P*-values and fold-changes of the consensus list miRNAs are shown in a colorimetric scale in Figure 2A and B respectively. Imposing a *P*<0.005 on all four platforms, no miRNAs were commonly DE. At *P*<0.05, hsa-miR-378, hsa-miR-375, hsa-miR21*, hsa-miR-145 were detected as DE by all platforms and a further 4 miRNAs (hsa-miR-96, hsa-miR21, hsa-miR147b, and hsa-miR-143) were DE on all but one platform; in fact, on the Miltenyi platform, hsa-miR-96 and hsa-miR-147b were not detected, while hsa-miR-21 and hsa-miR-143 did not reach a significant threshold. Twelve, 2, and 25 miRNAs were found to be exclusively DE on the Agilent, Exiqon, and Illumina platforms, respectively. The remaining 29 miRNAs were DE in at least two platforms. The fold changes are concordant across platforms with the only exception of two miRNAs (hsa-miR-218 and hsa-miR-302a) that were DE at *P*<0.05 in Illumina and Exiqon, but with discordant fold-changes (Fig. 2B).

In order to verify that the limited number of commonly DE miRNAs was not a result of the normalization methods, we calculated the number of differentially expressed miRNAs in each platform and for each of the four normalization methods. For the 256 ( = 4^4^) possible combinations, we identified a list of shared DE miRNAs. The union of all these lists gathered four miRNAs (hsa-miR-378, hsa-miR-375, hsa-miR-145, hsa-miR-21*), suggesting that different normalization methods can be worse than or, at best, equal to our choice (Fig. S4A). Noteworthy, among the 4 common miRNAs the hsa-miR-378 was identified in all the possible combinations (Fig. S4B).

**Figure 3:**
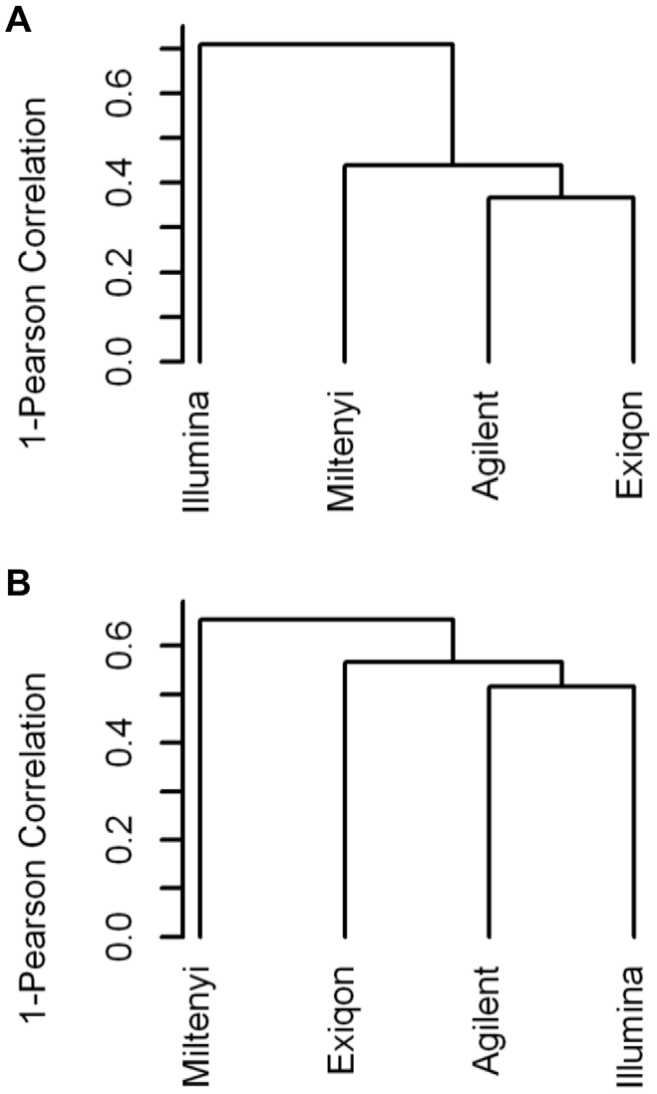
Clustering analysis of log2 fold changes and *t*-values. Hierarchical clustering (distance = Pearson correlation; linkage = average) of log2 fold changes (A) and t-values (B) obtained for each platform by comparing tumor and normal samples in the subgroup of commonly detected miRNAs. t-values were calculated using a t-test with random variance model.

The overall platform comparability in terms of accuracy and ability to identify DE miRNAs was evaluated focusing respectively on fold changes and t-values obtained in the tumor/normal comparison for the 233 miRNAs commonly detected by the 4 platforms. After clustering analysis, the best correlation among log_2_ fold changes were observed between Agilent and Exiqon (Pearson’s correlation = 0.63), whereas Illumina showed the most different pattern and wider fold changes (Fig. 3A and Fig. S5A). In the same way, only a partial similarity in t-values (Pearson’s correlation; range = 0.28–0.48; average = 0.40) is present among the 4 platforms, but this time Miltenyi showed the most divergent behavior (Fig. 3B and Fig. S5B).

### Inter-platform Agreement using miRNA Sets

**Figure 4:**
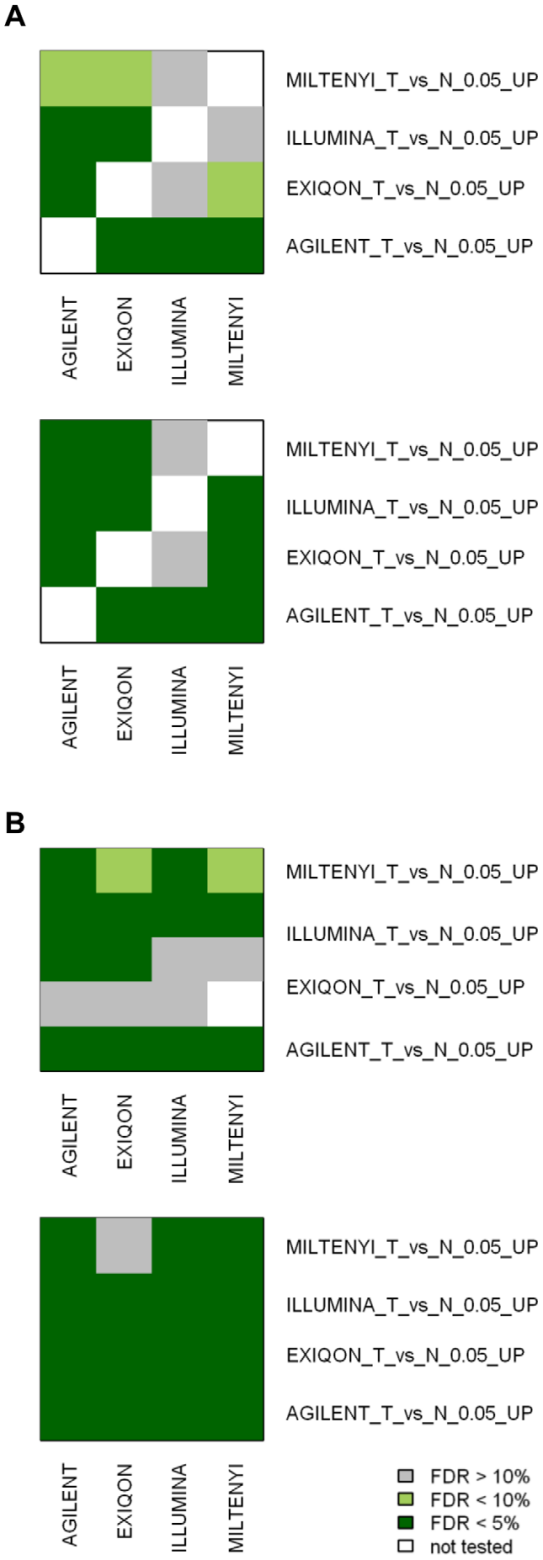
miRNA set enrichment analysis. Summary of miRNA set enrichment analysis performed using GSEA. Using the expression data obtained with the 4 different platforms, we tested the enrichment of miRNAs DE (when comparing colorectal cancer and normal mucosa) in our study (A) or reported in the literature (B). miRNAs up- or down-regulated were tested separately. For the literature-derived miRNA sets, the firs author and the platform used were indicated (see also Table S3). False Discovery Rates less than 5% or 10% were considered significant or marginally significant respectively.

Previous studies comparing the performance of gene expression microarray platforms suggested that, despite a relatively low overlap among lists of DE genes was obtained with different platforms, a good agreement was found when looking at biologically related gene sets instead of single genes [14]. To test whether similar conclusions can be drawn for miRNA microarray platforms, we performed a miRNA set enrichment analysis on our data testing two series of miRNA sets: 1) the DE miRNAs identified by each platform in our study, to evaluate their enrichment among up or down-regulated miRNAs on the other platforms; 2) miRNAs identified as up- or down- regulated between colon cancer and normal mucosa in other microarray based studies from the literature (Table S3). Most of the miRNA sets identified by each platform are coherently enriched in data from the other platforms, with the Miltenyi miRNA set showing the lower enrichments (Fig. 4A). Moreover, the great majority of colon cancer associated miRNA sets derived from the literature were also validated in our data and, at least in part, independently of the tested platform (Fig. 4B).

### Comparison with qRT-PCR Data

Microarray data are regularly validated by qRT-PCR. Different systems are commercially available and, as pointed out for the microarray platforms, qRT-PCR manufacturers also have to deal with the continuous update of miRBase annotations. As a validation method, depending on the availability of selected miRNA assays at the time the experiments were performed, SYBR Green LNA assays from Exiqon or Applied Biosystem Taqman assays were used.

We focused our validation analysis on 18 miRNAs that summarize different situations found in the platform comparison (Table 2). The 8 DE miRNAs in at least 3 of 4 array platforms were validated as significantly DE by qRT-PCR. For these 8 miRNAs, high correlations between qRT-PCR and array expression values and in pair-wise contrasts of array data were observed (Table 3 and File S1) with two exceptions; in the case of hsa-miR-21*, although qRT-PCR data confirmed the differential expression found in all array platforms, its correlation with array data was limited (R coefficient’s range 0.27–0.44); for hsa-miR-21, the values on Illumina did not correlate with any other values obtained on arrays or by qRT-PCR. This latter discrepancy is likely attributable to the miR-21 expression values on Illumina that are near to saturation in all samples and, for this reason, concentrated in a limited range.

**Table 2:** miRNA arrays and qRT-PCR class comparison.

			Class comparison tumor/normal									
			qPCR		Agilent		Exiqon		Illumina		Miltenyi	
		miRNA	FC	p-val	FC	p-val	FC	p-val	FC	p-val	FC	p-val
Differentially expressed in at least 3/4 platforms	Concordant	hsa-miR-378	0.18	0.0000	0.49	0.0002	0.40	0.0000	0.40	0.0003	0.67	0.0130
		hsa-miR-375	0.14	0.0009	0.40	0.0005	0.70	0.0055	0.55	0.0441	0.57	0.0337
		hsa-miR-21*	1.54	0.0254	1.64	0.0460	1.32	0.0009	1.82	0.0009	1.47	0.0086
		hsa-miR-145	0.10	0.0065	0.30	0.0027	0.49	0.0456	0.68	0.0019	0.35	0.0184
		hsa-miR-96	3.73	0.0008	1.77	0.0050	1.18	0.0428	4.43	0.0024		
		hsa-miR-21	1.86	0.0118	2.47	0.0033	2.11	0.0159	1.11	0.0081	1.42	0.1709
		hsa-miR-147b	0.12	0.0015	0.83	0.0000	0.81	0.0170	0.39	0.0005		
		hsa-miR-143	0.17	0.0118	0.42	0.0437	0.30	0.0364	0.69	0.0018	0.47	0.1252
Differentially expressed in at least 2/4 platforms	Concordant	hsa-miR-93	0.84	0.4667	1.61	0.0202	1.17	0.0790	1.36	0.0050	1.20	0.2097
		hsa-miR-886-5p	2.41	0.0130	1.02	0.3686			1.73	0.0189	1.48	0.0002
		hsa-miR-886-3p	0.93	0.7370	1.09	0.0004			1.25	0.0956	1.88	0.0002
		hsa-miR-497	0.28	0.0051	0.61	0.0008			0.35	0.0060	0.81	0.1473
		hsa-miR-30a	0.27	0.0016	0.46	0.0002	1.32	0.2676	0.50	0.0015		
		hsa-miR-182	3.60	0.0012	1.04	0.0256	1.10	0.4681	2.65	0.0000		
		hsa-miR-139-5p	0.10	0.0010	0.79	0.0025	0.76	0.0664	0.17	0.0023	0.77	0.2078
		hsa-miR-136	0.23	0.0153	0.69	0.0037	0.98	0.7852	0.48	0.0176		
	Discordant	hsa-miR-218	0.19	0.0410	0.96	0.3632	1.22	0.0363	0.34	0.0020		
		hsa-miR-302a	0.95	0.8335	1.02	0.3413	1.20	0.0247	0.54	0.0020		

FC = fold change.

To better understand the basis of the poor overlap of class comparison results in the four platforms, we measured the expression of 10 further miRNAs (Table 3 and File S1).

**Table 3:** Pair-wise correlations.

			Pearson correlation analysis									
		miRNA	qPCR vs Agilent	qPCR vs Exiqon	qPCR vs Illumina	qPCR vs Miltenyi	Agilent vs Exiqon	Agilent vs Illumina	Agilent vs Miltenyi	Exiqon vs Illumina	Exiqon vs Miltenyi	Illumina vs Milteny
Differentially expressed in at least 3/4 platforms		hsa-miR-378	0.67	0.87	0.83	0.54	0.64	0.63	0.75	0.9	0.59	0.68
		hsa-miR-375	0.91	0.7	0.86	0.49	0.67	0.85	0.32	0.63	0.86	0.52
		hsa-miR-21*	0.44	0.42	0.39	0.27	0.1	0.16	0.39	0.52	0.64	0.57
		hsa-miR-145	0.94	0.81	0.96	0.83	0.78	0.92	0.81	0.81	0.96	0.84
		hsa-miR-96	0.53	0.48	0.67	–	0.74	0.69	–	0.6	–	–
		hsa-miR-21	0.76	0.77	−0.05	0.72	0.93	0.00	0.87	−0.23	0.87	0.02
		hsa-miR-147b	0.67	0.55	0.72	–	0.47	0.76	–	0.61	–	–
		hsa-miR-143	0.66	0.68	0.86	0.59	0.91	0.6	0.88	0.64	0.88	0.57
Differentially expressed in at least 2/4 platforms	Concordant	hsa-miR-93	0.33	−0.25	−0.19	−0.14	0.07	0.5	−0.07	0.4	0.61	0.44
		hsa-miR-886-5p	0.15	–	0.76	0.26	–	0.25	0.27	–	–	0.21
		hsa-miR-886-3p	0.41	–	0.48	0.00	–	0.69	0.71	–	–	0.4
		hsa-miR-497	0.79	–	0.79	0.52	–	0.84	0.65	–	–	0.63
		hsa-miR-30a	0.7	−0.01	0.65	–	−0.47	0.46	–	−0.2	–	–
		hsa-miR-182	0.48	0.17	0.86	–	0.55	0.58	–	0.04	–	–
		hsa-miR-139-5p	0.83	0.76	0.87	0.27	0.63	0.79	0.22	0.74	0.91	0.48
		hsa-miR-136	0.69	0.29	0.72	–	0.25	0.56	–	0.31	–	–
	Discordant	hsa-miR-218	0.5	−0.41	0.77	–	0.06	0.55	–	−0.32	–	–
		hsa-miR-302a	0.24	−0.01	0.06	–	−0.2	−0.04	–	−0.52	–	–

Six of them (hsa-miR-136, hsa-miR-139-5p, hsa-miR-182, hsa-miR-30a, hsa-miR-497, and hsa-miR-93) were selected among the 14 DE miRNAs (*P*<0.05) according to both Agilent and Illumina. We validated the array data by qRT-PCR for 5 of these 6 miRNAs, with the relevant exception of hsa-miR-93. Correlation coefficients between qRT-PCR and either Agilent or Illumina data ranged from 0.65 to 0.87 for hsa-miR-136, hsa-miR-139-5p, hsa-miR-30a, and hsa-miR-497; for hsa-miR-182, whose probe intensities on Illumina were at intermediate levels and DE at *P*<0.005 and on Agilent were near to the background and DE at *P*<0.05, were 0.86 and 0.48, respectively.

Two other miRNAs, hsa-miR-886-5p and hsa-miR-886-3p, selected for qRT-PCR validation were concordant in 2 of the four platforms. The differential expression of hsa-miR-886-5p, DE on Illumina and Miltenyi platforms, was confirmed by RT-qPCR, while, that of hsa-miR-886-3p, DE on Miltenyi and Agilent platforms, did not appear to be DE by qRT-PCR.

Finally, we selected two miRNAs (hsa-miR-218 and hsa-miR-302a) that were DE on Exiqon and Illumina platforms but with opposite fold changes. hsa-miR-218 reduced expression in tumors on Illumina was confirmed by qRT-PCR while that of hsa-miR-302a was not validated using qRT-PCR.

**Figure 5:**
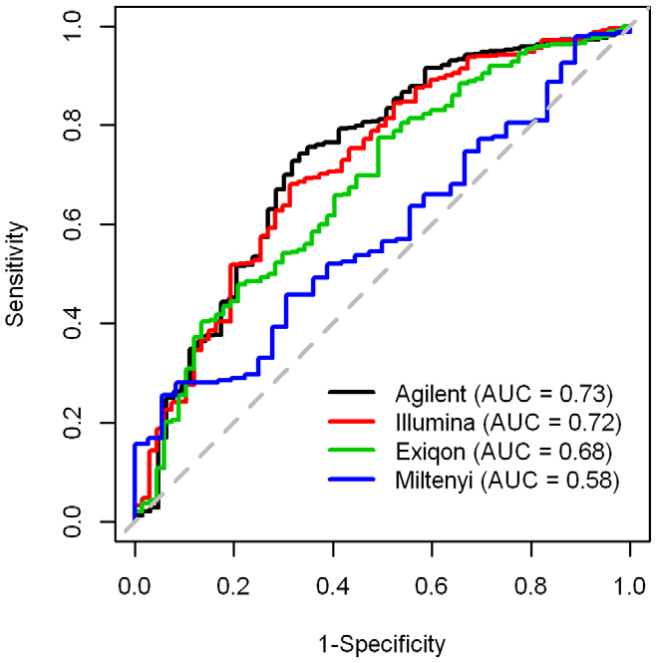
Performance assessment of the platforms. Considering as gold standard the miRNAs identified as differentially expressed in a qPCR study on 40 paired tumor-normal samples, we evaluated the performance of each platform calculating sensitivity and specificity at different thresholds of *P*-value and plotting the resulting values in the ROC space.

Real time PCR data are generally used to determine the sensitivity and specificity of data obtained with microarrays. To this aim, we compared our results to those obtained in an independent published qRT-PCR study, in which 70 of 665 unique miRNAs tested were found differentially expressed in 40 paired normal-colon cancer samples [15]. For each platform we selected miRNAs present in the qPCR dataset (527 for Agilent, 596 for Illumina, 545 for Exiqon and 278 for Miltenyi) and computed ROC curves using different thresholds of *P*-value. (Fig. 5). The values of Area Under the ROC Curve (AUC) showed that Agilent and Illumina are very similar and are the most accurate platforms while Miltenyi is the less performing.

### Biological Insight

When the 68 miRNAs DE at *P*<0.005 in at least one of the four platforms were compared with literature data, we found that 25% of them were concordantly described in literature as deregulated in colorectal cancer in comparison to the non tumor counterpart (Table S4). Furthermore, we found that 12 miRNAs belong to known co-expressed family clusters. The main biological data associated to the four miRNA clusters are reported in table 4. Looking at their expression we observed that: for miR 25–106b cluster, only hsa-miR-25 and hsa-miR-93 are present in the list of 68 miRNAs at the thresholds we applied; the miR 182-96 cluster is particularly evident in Illumina where hsa-miR-182, −182*, −183, and −96 are among the most up-regulated miRNAs in this platform (fold changes tumor vs normal ranging from 4.42 to 2.65); the miRNA cluster 143–145 is coherently deregulated in all the four platforms of our study, being hsa-miR-143 the most down-regulated miRNA in tumor tissues on Exiqon platform (fold change tumor vs normal tumor = 0.30; p = 0.036) and hsa-miR-145 the most down-regulated in Agilent and Miltenyi (fold change tumor vs normal = 0.30 and 0.35; p = 0.0027 and 0.018 respectively).

**Table 4:** Role in colon cancer of miRNA clusters DE in our study.

miRNA cluster	members	Chromosome location	Role in colon cancer	Reference
miR 195–497	hsa-miR-195hsa-miR-497	17p13.1	Chromosomal region frequently deleted in colorectal cancer.hsa-miR-195 is associated to lymph node metastasis, advanced tumor stage, and pooroverall survival.	[35] [36]
miR 25–106b	hsa-miR-25hsa-miR-93hsa-miR-106b	7q22.1	hsa-miR-25 is associated with lymphatic andvenous invasion,a more aggressive tumor phenotype.This cluster is closely relatedwith oncomir1.	[37]
miR 182-96	hsa-miR-182 hsa-miR-182* hsa-miR-183 hsa-miR-183* hsa-miR-96	7q32.2 intergenicregion	Not reported; in medulloblastoma this cluster promotes tumorigenesis regulating cellular migration.	[38]
miR 143–145	hsa-miR-143 hsa-miR-145	5q32	Altered expression is reported.This cluster is associated with negativeregulation on cell proliferation	[39]

**Figure 6:**
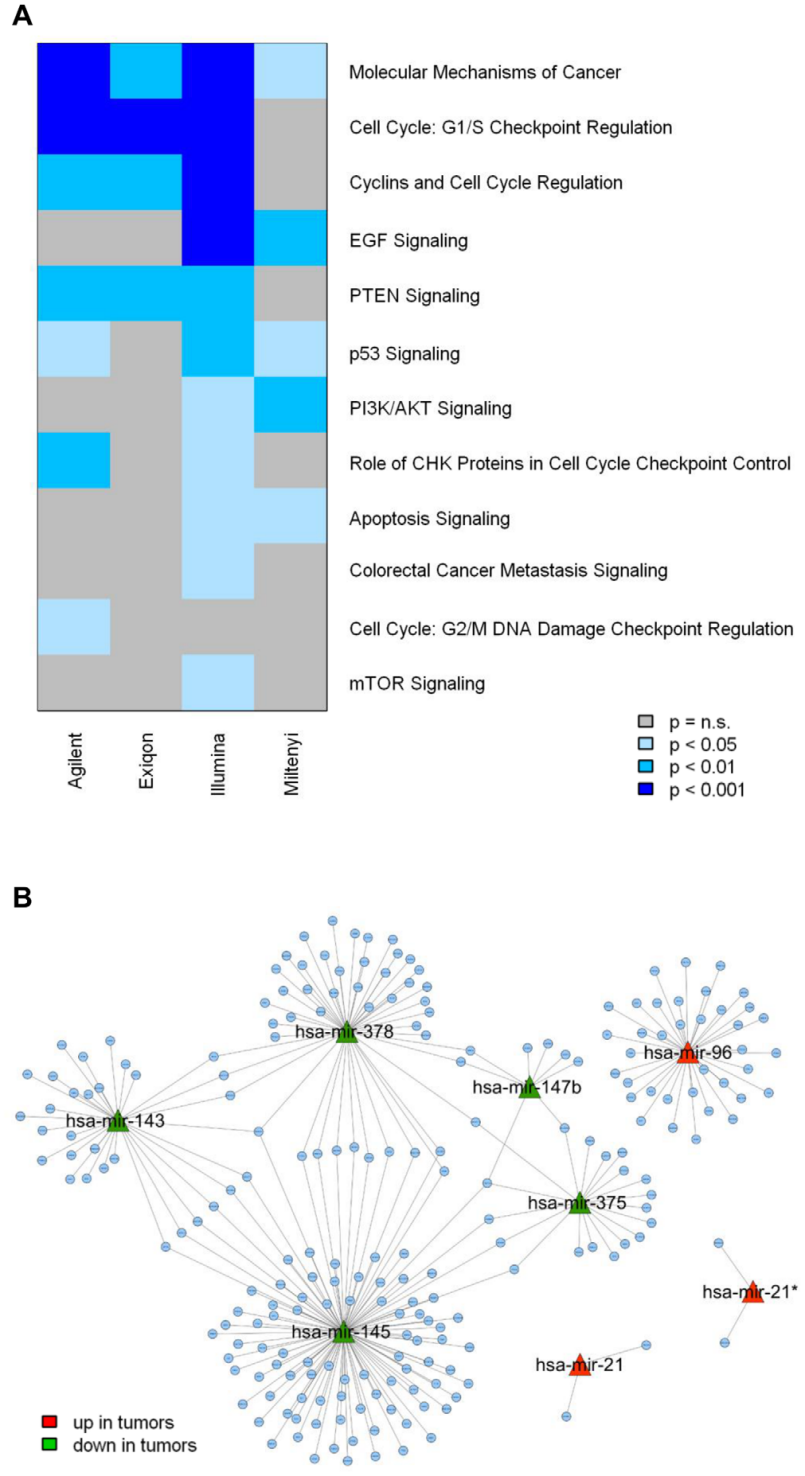
Computational integration of miRNA and gene expression profiles of the paired tumor/normal colon samples. (A) Pathway enrichment analysis of anti-correlated predicted target genes of differentially expressed miRNAs according to each microarray platform. (B) Network between the top 8 differentially expressed miRNAs and their anti-correlated target genes. The 250 top interactions were used to generate the network using MAGIA tool.

Gene expression profiles of the same samples analyzed by miRNA expression arrays were available. Thus, we considered an integration approach to evaluate whether similar biological information could be retrieved from the four platforms, irrespectively of the overlap in DE miRNAs. To this aim, using the MAGIA tool, negatively correlated putative target genes of DE miRNAs were identified in each platform (File S2) and an enrichment analysis was performed by IPA software. To highlight the concordance among the four platforms, enrichment *P*-values for all the cancer-related pathways significantly enriched in at least one platform are shown in a colorimetric scale in Figure 6A. Pathways related to cell cycle regulation and PTEN signalling were concordantly identified. When we looked at validated targets by TarBase software, the number of miRNA-mRNA interactions negatively correlated at p<0.05 was very limited (Agilent = 35, Exiqon = 2, Illumina = 45 and Miltenyi = 0) precluding a comparison across the four platforms.

Furthermore, by considering the qRT-PCR data of the 8 most concordant miRNAs and the gene expression profiles, the same integration approach identified a total of 803 miRNA-negatively correlated gene (predicted as miRNA targets) interactions (File S2). The graphical representation of the top 250 interactions highlighted that many genes that were up-regulated in tumors are predicted targets of two or more down-regulated miRNAs (Fig. 6B). In detail, there are 70 genes co-targeted by at least two miRNAs and 84% of them are regulated by miR 143–145 cluster (Table S5). Among these genes those related to glycolysis and nutrient transport pathways seemed over-represented.

## Discussion

Despite their relatively recent discovery, there is a rapidly growing interest in the study of the role of miRNAs in many pathological processes including cancer. Accordingly, high throughput technologies, initially developed for GW gene expression evaluation, were rapidly adapted to GW measurement of miRNAs. However, as highlighted in recent reviews [5]; [16]; [17], several factors, including short miRNA length, high degree of homology in miRNA families, the high rate of new miRNA identification (the actual number of miRNAs in miRBase 18, released in November 2011 is approaching two thousands) and the relatively high percent (about 10%) of artefactual miRNAs not confirmed by resequencing experiments, significantly complicate their analysis. The impact of these factors on the different methodologies applied by manufacturers of different available platforms must be considered in inter-platform comparison studies.

The issues of intra- and inter- microarray platform reproducibility have been mainly addressed using experimental settings where tissues or cell lines of different origin are compared, with the assumption that, due to the wide range of expected expression modulations by such comparison, technical noise can become negligible. This type of approach mirrored the one followed in its first phase study by the MicroArray Quality Control (MAQC) consortium, aiming to assess the inter-platform and inter-laboratory reproducibility of gene-expression microarray data using two different RNAs (human brain and a universal human reference) [18]. This approach was strongly questioned in 2007 for its lack of consistency with real research settings [19]. However, in the majority of miRNA inter-platform comparison studies, quoted in Aldridge & Hadfield [16] and reported in Table S1, the experimental design was biased toward the use of samples with strong difference in origin. Noteworthy, only two studies [7]; [10] compared the miRNA profile of biological meaningful samples on, at least, three different platforms, but even in these cases the samples are cell lines. Thus, our study represents the first attempt to compare miRNA platform performance in a clinical setting, where the inter-sample variability within the same class is expected to be higher than in cell lines.

The majority of profiling studies using clinical samples aimed at revealing even subtle differences in expression but which are associated to a specific clinical context. In these settings, technical replicates are frequently not feasible due to RNA quantity and economical considerations. Thus, in the present study we addressed the issue of inter-platform comparison using samples belonging to two classes (paired tumor and normal colon tissues) which could theoretically lead to new insights in tumor biology and clinical applications. Our data, generated by profiling the same tissue-derived total RNAs using four different miRNA array platforms, showed little overlap between platforms except for a limited number of miRNAs for which very high correlations were observed. These data are essentially in agreement with those obtained using cell lines since also in these studies only few miRNAs were shared among all platforms [7]; [10].

The first issue we considered was the global distribution of the hybridization intensities. The Illumina platform showed the most diverging behavior in global distribution of intensities compared to the other three platforms. An explanation could be the amplification step of the starting material, according to the Illumina protocol, while for the other platforms direct labeling of the starting material is performed. The amplification step allows the detection of a higher number of miRNAs expressed at low levels (e.g. hsa-miR-182), but with the drawback that it can lead to saturation of signals for more abundant miRNAs such as hsa-miR-21, which is expected to be both biologically and clinically relevant in many cancer types including colorectal cancer [20]. Due to the withdrawal of the platform, the saturation of signals remains a note of caution for former Illumina users.

The short length of miRNAs, their variable GC content, and the existence of families of miRNAs differing in one or only few nucleotides pose a set of technical challenges that each manufacturer has attempted to overcome through *ad-hoc* approaches. An evaluation of the GC content of detected and undetected probes in each platform confirmed the relevance of this parameter in determining the detection performance of all of them, but also highlighted that the Miltenyi platform is exceedingly sensitive to GC content, partially explaining its low detection rate.

In class comparison analysis between tumor and normal samples, much more modulated miRNAs were identified on Agilent and Illumina platforms compared to the few identified on the Exiqon and Miltenyi platforms. In Exiqon data, most of miRNAs modulated in Agilent and Illumina were detectable, although they did not reach statistical significance; on the other hand, the same miRNAs were frequently undetected on the Miltenyi platform. Focusing on the 233 commonly detected miRNAs, Miltenyi clustered separately from the other three platforms considering t-values, while Illumina shows the worst correlations with the others three platforms when considering fold-changes. qRT-PCR is frequently used as a “gold standard” to corroborate data using microarrays, but, as previously reported by others [7]; [17], qRT-PCR might also perform poorly in measuring some miRNAs, thus challenging its role as a “gold standard”. Moreover, the validity of qRT-PCR as a reference technique requires the application of superior standards to ensure its validity and the adherence to MIQE, i.e. the specific guidelines for minimum information for publication of quantitative real time PCR experiments [21]. Thus, in our analysis, we decided to use this technique, as generally done in a clinical setting, selecting only a small subset of miRNAs. It is worthwhile noting that all the 8 miRNAs concordantly DE on at least 3 of the 4 platforms were confirmed as DE by qRT-PCR, while in regard to the other 10 miRNAs assessed by qRT-PCR, 7 were validated.

Furthermore, since previous studies suggested that, despite a relatively low overlap among lists of DE genes obtained with different platforms, a higher agreement could be obtained looking at biologically related gene sets instead of single genes [14], we performed a miRNA set enrichment analysis on our data. In this case, we were able to appreciate a better inter-platform agreement compared to an approach based on single miRNA. In addition, a coherent enrichment was found for miRNA sets obtained from literature even using platforms different from the four analyzed in our study.

Undoubtedly, technical and analytical challenges in measuring miRNAs still remain and further research is required in order to increase consistency between different microarray-based methodologies. Overall, the poor inter-platform comparability seems to be reasonably due to a high false negative rate, with some probes performing poorly; among the four tested platforms, Illumina and Agilent, due to their high throughput performance, to the good concordance with qRT-PCR for the most DE miRNAs, and to the good sensitivity/specificity by ROC curves, resulted adequate for miRNA GW evaluation of clinical specimens. Finally, comparison studies could be relevant to other researchers not only in making the proper decision regarding the best platform to use in their projects but also for a better interpretation of their results.

Looking at literature data we found that some miRNAs, identified as DE in our study, have been already implicated in colon cancer development and progression (see also comments and references in Table 4 and Table S4). Noteworthy the miRNA more up-regulated in tumor samples in two platforms (Agilent and Exiqon; fold change tumor vs normal = 2.47 and 2.1; p = 0.0033 and 0.0159 respectively) is hsa-miR-21, that represents a well-established pro-oncogenic miRNA in many tumors including colorectal cancer [22]. In addition, hsa-miR-378 was identified in other screenings investigating the differential miRNA expression in normal and neoplastic colon tissues [23], and it is worth to note that in our qRT-PCR data its expression levels in normal and tumor samples were not overlapping (File S1) making it a promising candidate as diagnostic marker.

miRNAs regulate gene expression by triggering either repression of translation or mRNA degradation [24]; [25]. An integrated approach to better understand the relationship between miRNA and mRNA is often used in order to gain insight into miRNA function. Following this approach, we evaluated the biological information provided by each platform through the integration with the gene expression profile available for the same samples. As proof of principle, target genes, negatively correlated to the miRNAs modulated according to each platform, were commonly enriched in cancer related pathways. In principle, such analysis restricted to the validated targets could be more informative. However, at present, the number of validated targets is quite small compared to that of predicted ones, not only due to the intrinsic limits of the applied algorithms but also for the experimental complexity of the validation process. In our datasets the number of validated targets is low and the models in which the predictions were validated are far from the clinical context of our interest. All together these limitations precluded further analyses. Noteworthy, some genes were co-targeted by two or more miRNAs and among them we noticed the presence of genes related to glycolysis and nutrient transport pathways. As a matter of fact, the gene-miRNA pair predictions point out that expression of both hsa-miR-143 and hsa-miR-145 inhibits Hexokinase 2 (HK2) expression. HK2 is the first rate-limiting enzyme of glycolysis, conferring to the tumor an increased proliferation capacity and invasiveness when expressed at elevated levels [26]; [27]. In this context, we found other predicted target genes negatively correlated with members of miRNA 143–145 cluster as involved in glycolysis nutrient transport pathways, such as GLUT1/SLC2A1, a key gene required for glucose uptake with an important role in carcinogenesis [28] and HCP1/SLC46A1, a proton-coupled folate transporter, important in intestinal folate absorption [29]. In addition, three members of solute carrier family 7, a family of amino acid transporters, SLC7A1, SLC7A11 and SLC7A6, negatively correlated with miRNA 143–145 cluster, are reported as up-regulated in tumor cells due to the demand for increased amino acid transport during cancer progression [30]; [31].

In conclusion, our study does not aim at advertising any platform, since each has inherent upsides and downsides. Despite the identified limits, our analysis allowed the identification of a concordantly set of miRNA clusters deregulated between tumor and normal colorectal tissues and through an integrative miRNA- gene expression analysis and the support of available literature, we were able to shed light on the biological role of these miRNAs and on their involvement in colorectal tumorigenesis.

Finally, the ever-increasing improvement in microarray and NGS designs and technologies are hopefully expected to allow a robust identification and validation of miRNAs as biomarkers.

## Materials and Methods

### Ethics Statement

All patients whose biological samples were included in the study signed an informed consent, approved by the Independent Ethical Committee of the Fondazione IRCCS Istituto Nazionale dei Tumori Milano (INT-MI), to donate to INT-MI the leftover tissue specimens after completing diagnostic procedures for research purposes. The Independent Ethical Committee of INT-MI approved the use of the samples for this specific study in the framework of a project in biobanking quality control.

### Tumor Samples and RNA Extraction

Tumor and normal matched samples were prospectively collected from 9 patients who underwent surgical resection at INT-MI. Histological and clinical characteristics of samples are listed in Table S2. Neoplastic samples were obtained from the central area of the tumor, avoiding necrotic material or transition zones with healthy mucosa. Samples of colonic healthy mucosa were collected at least 20 cm from the tumor and distant from surgical resection margins. Tissue samples were collected within 20 minutes after surgical resection and were stored at −80°C until RNA extraction in the frozen INT-MI Tissue Bank. Total RNA was extracted from 10–20 mg of tumor samples and from 30–40 mg of normal tissues. Samples were mechanically disrupted and simultaneously homogenized in the presence of QIAzol Lysis reagent (Qiagen, Valencia, CA, USA), using a Mikrodismembrator (Braun Biotech International, Melsungen, Germany). RNA was extracted using the miRNeasy Mini kit (Qiagen) according to manufacturer’s instructions. RNA concentrations were measured with the NanoDrop ND-100 Spectrophotometer (NanoDrop Technologies, Wilmington, DE), while RNA quality was assessed with the Agilent 2100 Bioanalyzer (Agilent Technologies, Palo Alto, CA) using the RNA 6000 Nano kit (Agilent Technologies). Samples included in the present analysis had a RIN score greater than 5 and a 28S:18S rRNA ratio close to 2∶1.

### miRNA Expression Profiling

The amount of input RNA, labeling, and hybridization conditions were chosen following the recommendations of each manufacturer. The experimental work was performed over a period of six weeks to minimize biases caused by environmental ozone levels.

Each manufacturer developed its procedures that involved dedicated protocols for labeling, equipment for hybridization and scanning, and software for data acquisition. Illumina chips were processed by the Functional Genomic core facility of INT-MI Department of Experimental Oncology and Molecular Medicine, whereas Agilent hybridizations were carried out at Fondazione Edo ed Elvo Tempia’s Cancer Genomics laboratory. In both cases, experiments were performed by qualified and trained personnel. Exiqon and Miltenyi slides were processed under specialist supervision from both companies by the INT-MI Functional Genomic core facility.

#### Agilent arrays

Agilent miRNA array analysis was carried out at Fondazione Edo ed Elvo Tempia Foundation according to the manufacture’s instructions. One hundred ng total RNA was dephosphorylated at 37°C for 30 min with calf intestinal phosphatase and denatured using 100% DMSO at 100°C for 5 min. Samples were labeled with pCp-Cy3 using T4 ligase by incubation at 16°C for 1 hour and hybridized on a 8_x_15K format Agilent human miRNA array. Arrays were washed according to manufacturer's instructions and scanned at a resolution of 5 µm using an Agilent 4000B scanner. Data were acquired using Agilent Feature Extraction software version 9.5.3.1.

#### Illumina arrays

Mature miRNAs were amplified with the Illumina human_v2 MicroRNA expression profiling kit based on the DASL (cDNA-mediated Annealing, Selection, Extension, and Ligation) assay, according to the manufacturer’s instructions. Briefly, 600 ng total RNA was converted to cDNA and annealed to a miRNA-specific oligonucleotide pool consisting of three parts: a universal PCR priming site at the 5′ end, an address sequence complementary to a capture sequence on the BeadArray, and a miRNA-specific sequence at the 3′ end. After PCR amplification and fluorescent labeling, the probes were hybridized on Illumina miRNA BeadChips. After hybridization and washing, fluorescent signals were detected by the Illumina BeadArrayTM Reader. Primary data were collected using V3.1.3.0 software.

#### Exiqon arrays

miRNA expression profiling was conducted with the use of 0.3 µgtotal RNA that were labeled with Cy3 fluorescent dye, using the miRNA/LNA labeling kit (Exiqon, Vedbæk, Denmark). The fluorescently labeled samples were hybridized to a miRNA microarray using a GeneTac hybridization station. The microarray slides were scanned with GenePix 4100 scanner (Axon Instruments, Union City, CA) and raw data were collected with GenePix 6.0.

#### Miltenyi arrays

Labeling and hybridization were performed according to user manuals of the miRXplore™ instrument (Miltenyi Biotec, Bergisch-Gladbach, Germany). In brief, 1.2 µg/sample total RNA was labeled with the red fluorescent Hy5 using the miRNA/LNA labeling Exiqon kit. A pool of synthetic miRNAs in equimolar concentrations was designed by Miltenyi based on sequences of miRBase 9.2 and were labeled with Hy3. Subsequently, the labeled material was hybridized overnight to miRXplore™Microarrays using the a-Hyb™ Hybridization Station (Miltenyi Biotec, Bergisch-Gladbach, Germany). Fluorescence signals of the hybridized miRXplore™ Microarrays were detected using GenePix 4100 scanner and raw data were acquired with GenePix 6.0.

### miRNA Microarrays Data Processing and Statistical Analysis

Both Agilent and Illumina provide proprietary instruments for scanning the arrays (Illumina’s BeadArray Reader and Agilent Microarray Scanner) and software to assess the signal values, and define a qualitative detection call for each probe. Exiqon and Miltenyi are more flexible since their slides can be adapted to different scanners. Miltenyi and Exiqon image acquisition was carried out using a GenePix Axon scanner.

Images were visually inspected to remove artifacts. Raw data were corrected for background noise using the backgroundCorrect module present in limma R package, and spots with lower 30% of pixels with intensities more than one standard deviation above the background intensity were flagged. Agilent and Miltenyi data required an additional adjustment using ComBat [32] to correct a slight batch effect due to the day processing of the slides (Fig. S6).

Four different normalization procedures (loess, quantile, rank invariant, and RSN) were tested using the corresponding functions from the lumi R package [33].

All microarray data are MIAME compliant and the raw data were deposited into the NCBI’s Gene Expression Omnibus (GEO) database (http://www.ncbi.nmlm.nih.gov/projects/geo/) with the following accession numbers: Agilent GSE33124, Exiqon GSE33122, Illumina GSE33125, Miltenyi GSE33123.

### mRNA Expression Profiling and Normalization

RNA samples were processed for mRNA microarray hybridization by the INT-MI Functional Genomics core facility. Briefly, 800 ng of total RNA was reverse transcribed, labeled with biotin and amplified overnight (14 hours) using the Illumina RNA TotalPrep Amplification kit (Ambion, Austin, Texas, USA) according to manufacturer’s protocol. One µg of the biotinylated cRNA sample was mixed with the Hyb E1 hybridization buffer containing 37.5% (w/w) formamide and then hybridized to a Sentrix Bead Chip HumanHT12_v3 (Illumina, Inc., San Diego, CA) at 58°C overnight (18 hours). The array represents over 48,000 bead types, each with a unique sequence derived from human genes in the National Centre for Biotechnology Information Reference Sequence or UniGene database. Array chips were washed with the manufacturer’s E1BC solution, stained with 1 µg/ml Cy3-streptavidine (Amersham Biosciences; GE Healthcare, Piscataway, NJ, USA) and eventually scanned with an Illumina BeadArray Reader. We collected primary data using the supplied scanner software and subsequent analyses were performed using the BeadStudio Version 3.1.3.0 software package. Intensity values of each hybridization were quality checked and the dataset was quantile normalized.

All microarray data are MIAME compliant and the raw data were deposited into the NCBI’s Gene Expression Omnibus (GEO) database (http://www.ncbi.nmlm.nih.gov/projects/geo/) with accession number GSE33126.

### miRNA Set Enrichment Analysis

Enrichment analysis in miRNA expression data was performed using GSEA (v. 2.0) [14]. miRNAs DE between colorectal cancer and normal mucosa in our data or in other microarray-based studies (Table S3) composed the miRNA sets we tested. Separate miRNA sets were generated for up- or down- regulated genes and a minimum of 5 miRNAs present in the data was required to perform the enrichment test. miRNA sets with a FDR<5% and <10% were considered significantly enriched and marginally enriched respectively.

### Real-Time Quantitative PCR

Primers were obtained from Exiqon (Vedbæk, Denmark) if available. Otherwise, assays were purchased from Applied Biosystems (Foster City, CA, USA).

qRT-PCR microRNA assays specific for hsa-miR-21* (Assay ID 204302), hsa-miR-30a (Assay ID 204791), hsa-miR-93 (Assay ID 204715), hsa-miR-96 (Assay ID 204417), hsa-miR-136 (Assay ID 204779), hsa-miR-139-5p (Assay ID 204037), hsa-miR-143 (Assay ID 204190), hsa-miR-145 (Assay ID 204483), hsa-miR-147b (Assay ID 204368), hsa-miR-375 (Assay ID 204362), hsa-miR-378 (Assay ID 204179), were purchased from Exiqon. qRT-PCR was performed using the miRCURY LNA™ Universal RT microRNA PCR system (Exiqon) and following the manufacturer’s instructions. Twenty ng total RNA were polyadenylated and reverse transcribed at 42°C (60 min) followed by heat-inactivation at 85°C (5 min) using a poly-T primer containing a 5′ universal tag. The resulting cDNA was diluted 80-fold and 8 µl used in 20 µl PCR amplification reactions as follows: 95°C for 10 min and then 40 cycles of 95°C for 10 sec, and 60°C for 60 sec. Normalization was performed with snord48 (Assay ID:203903).

Applied Biosystem’s TaqMan microRNA assays specific for hsa-miR-21 (Assay ID 397), hsa-miR-182 (Assay ID 2334), hsa-miR-218 (Assay ID 521), hsa-miR-302a (Assay ID 529), hsa-miR-497 (Assay ID 1043), hsa-miR-886-3p (Assay ID 2194), hsa-miR-886-5p (Assay ID 2193), were used to detect and quantify mature microRNAs on Applied Biosystems real-time PCR instruments in accordance with the manufacturer’s instructions. Starting from 4 ngtotal RNA first strand cDNA was synthesized using miR-specific stem-loop primers and the High-Capacity cDNA Reverse Transcription Kit (Applied Biosystems), reactions were run in a GeneAmp PCR 9700 thermocycler (Applied Biosystems) at 16°C for 30 min, 42°C for 30 min, and 85°C for 5 min. The RT products, PCR master mix containing TaqMan 2× Universal PCR Master Mix (No Amperase UNG), and 10× TaqMan assay in 20 µL were amplified as follows: 95°C for 10 min, 40 cycles of 95°C for 15 sec, and 60°C for 60 sec. Normalization was performed with the small nuclear RNA, RNU48 (Assay ID:1006).

miRNA expression levels were quantified using a sequence detection system (ABI Prism 7900HT; AppliedBiosystems) in duplicate, and threshold cycle (Ct) for each sample was determined. ABI SDS 2.4 software was used to recover the data and relative expression (referred to small nuclear RNA48) was calculated using the comparative ΔCt method.

### Class Comparison

Differentially expressed miRNAs between tumor and normal colon tissues were identified using a two-sample paired *t-*test with random variance model at nominal significance level of 0.005, unless otherwise specified as implemented in the Biometric Research Branch (BRB) ArrayTools (Version 3.8) developed by Dr. Richard Simon and the BRB-ArrayTools development team. The random variance *t-*test was selected to allow displaying differentially expressed miRNAs without assuming that all probes possess the same variance.

### Integration of miRNA and mRNA Expression Data

Integration of miRNA and mRNA data was performed using the freely available tool MAGIA [34]. The first step is the prediction of targets for the submitted miRNAs. In our analysis these were identified according to the TargetScan algorithm. Next, the correlation between each miRNA and its predicted targets was computed. All predicted targets with a correlation <−0.4 (File S2) were submitted to enrichment analysis using the Ingenuity Pathway Analysis software, and cancer-related canonical pathways with Fisher’s exact test *P*<0.05 in at least one platform were selected.

## Supporting Information

Figure S1 **Distribution of differential expression of miRNAs (log_2_ fold changes) dependent on the origin of analyzed samples.** Four publicly available datasets, obtained on Agilent platform were used for distribution comparison: GSE13860 and E-MTAB-96 datasets belonging to miRNA cross-platform comparison studies (Table S1); GSE21036 dataset, 28 paired primary prostate tumors and normal matched tissues profiled on Agilent v2.0 arrays, designed on miRBase release 10.1 [12]; GSE28700 dataset, 22 paired gastric cancers and normal matched tissues profiled on Agilent v1.0 arrays, designed on miRBase 10.1 [13].
(PDF)

Figure S2 **RLE plots.** For each platform, RLE plots were generated separately for normal (green) and tumor (red) samples before and after normalization with one of the four methods taken in account. To evaluate the similarity of RLE values distribution we compared the standard deviations of the median, 25- and 75-percentile.
(PDF)

Figure S3 **Comparison of differentially expressed miRNAs dependent on platform used.** The histograms of log *P*-value and FDR frequency of the differentially expressed miRNAs in tumor/normal class comparisons are reported.
(PDF)

Figure S4 **Impact of normalization on differential analysis.** The four platforms were normalized using four different methods (Loess, Quantile, Rank Invariant, RSN). For each of the 256 possible combinations, the number of commonly differentially expressed miRNAs was computed and reported in (A). For microRNAs commonly detected as DE in at least one of the 256 combinations, the number of times they were selected is plotted in (B).
(PDF)

Figure S5 **Common miRNA correlation.** Pairwise correlation of log_2_ fold changes (A) and t-values (B) of the 233 miRNAs commonly detected by all platforms. Pearson correlation (R) and the slope (m) estimated by linear regression are shown.
(PDF)

Figure S6 **Batch effect correction.** Moderated F-test (LIMMA package) was performed among classes defined by batches and F value distributions are plotted before and after applying the ComBat method [32] to both Agilent and Myltenyi expression data. The F threshold corresponding to a *P*<0.01 is plotted. After the correction, the number of miRNAs with a significant F values was reduced.
(PDF)

Table S1 Summary of cross-platform studies comparing more than three different platforms.
(DOCX)

Table S2 Clinical and pathological characteristics of patients.
(DOCX)

Table S3 miRNA sets of DE miTNAs between colorectal cancer andnormal mucosa available from literature.
(DOCX)

Table S4 miRNA chromosome location, relative expression and comparison with literature data.
(DOCX)

Table S5 List of the genes co-targeted by at least two miRNAs.
(DOCX)

File S1 Pearson correlations between arrays and qPCR data for 18 selected miRNAs.
(PDF)

File S2 Anti-correlated miRNA targets (R<−0.4) identified using the MAGIA tool for each of the 4 miRNA platforms and for qRT-PCR data.
(XLSX)
