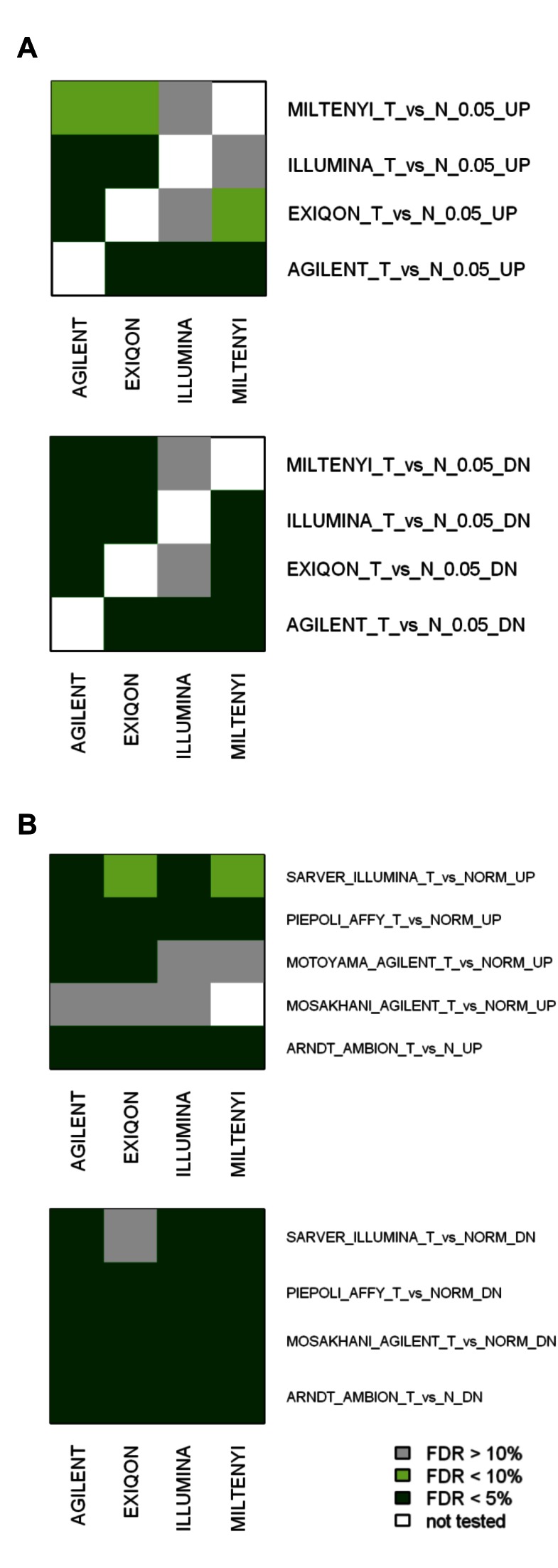# Correction: Comparison of Microarray Platforms for Measuring Differential MicroRNA Expression in Paired Normal/Cancer Colon Tissues

**DOI:** 10.1371/annotation/c38405cc-dc33-424e-bfcb-14de73f5c8c8

**Published:** 2013-05-14

**Authors:** Maurizio Callari, Matteo Dugo, Valeria Musella, Edoardo Marchesi, Giovanna Chiorino, Maurizia Mello Grand, Marco Alessandro Pierotti, Maria Grazia Daidone, Silvana Canevari, Loris De Cecco

There was an error in Figure 4. The correct version of the figure is available below.

Figure 4: 

**Figure:**