# Supplementary figures and images for: Comparison of Microarray Platforms for Measuring Differential MicroRNA Expression in Paired Normal/Cancer Colon Tissues
Source: PLoS One. 2012 Sep 13;7(9):e45105. doi: 10.1371/journal.pone.0045105 (PMC3441572; doi:10.1371/journal.pone.0045105)

**Figure S1**

**GSE13860**

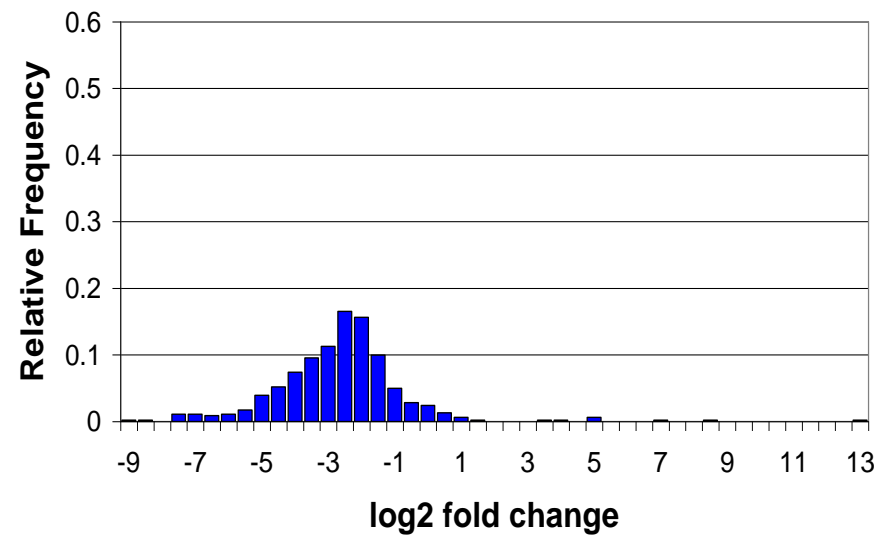

**E-MTAB-96**

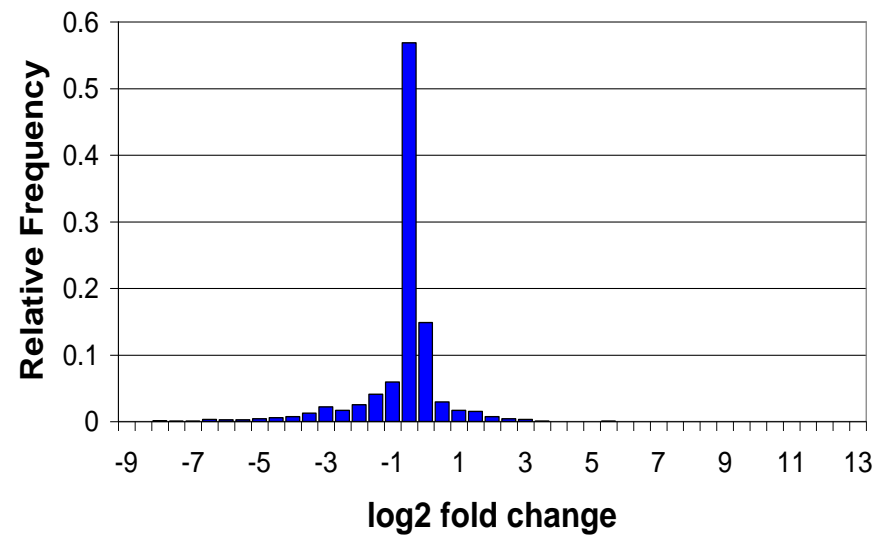

**GSE21036**

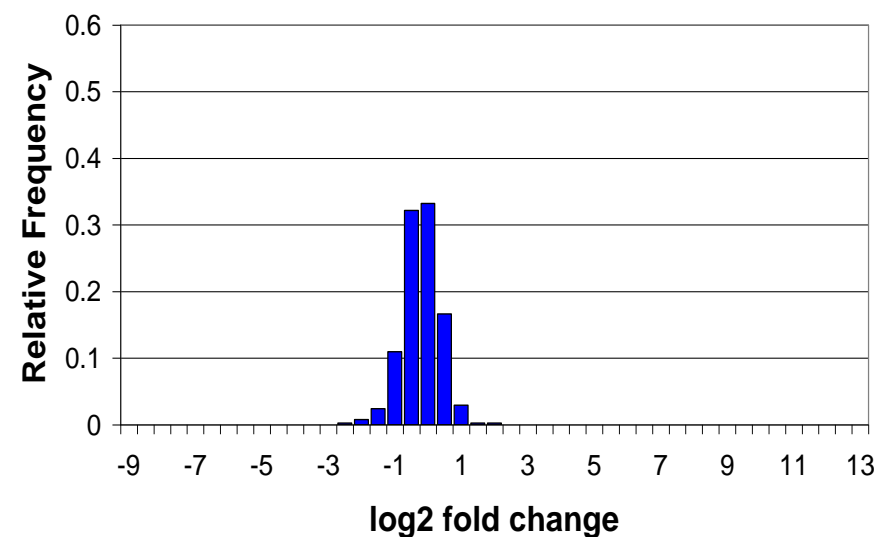

**GSE28700**

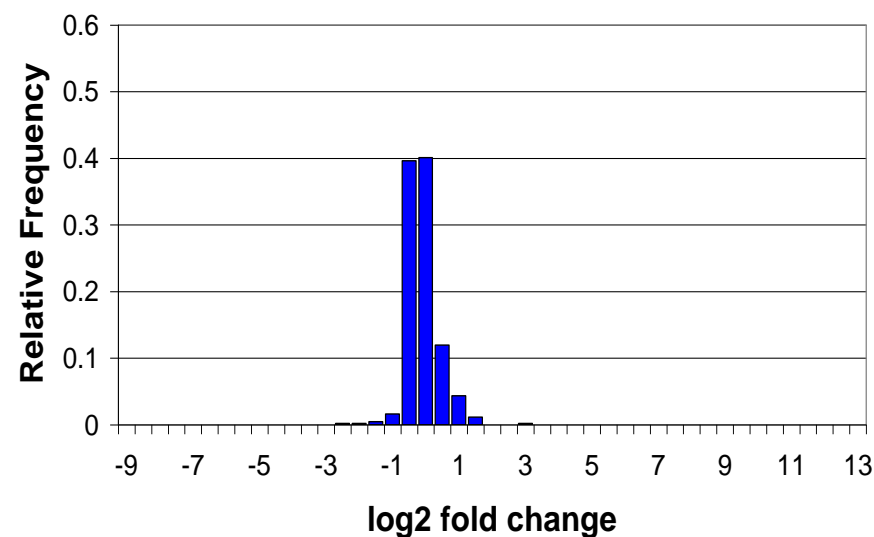

Supplement: Figure S1 — Distribution of differential expression of miRNAs (log2 fold changes) dependent on the origin of analyzed samples. Four publicly available datasets, obtained on Agilent platform were used for distribution comparison: GSE13860 and E-MTAB-96 datasets belonging to miRNA cross-platform comparison studies (Table S1); GSE21036 dataset, 28 paired primary prostate tumors and normal matched tissues profiled on Agilent v2.0 arrays, designed on miRBase release 10.1 [12]; GSE28700 dataset, 22 paired gastric cancers and normal matched tissues profiled on Agilent v1.0 arrays, designed on miRBase 10.1 [13]. (PDF) [file pone.0045105.s001.pdf]

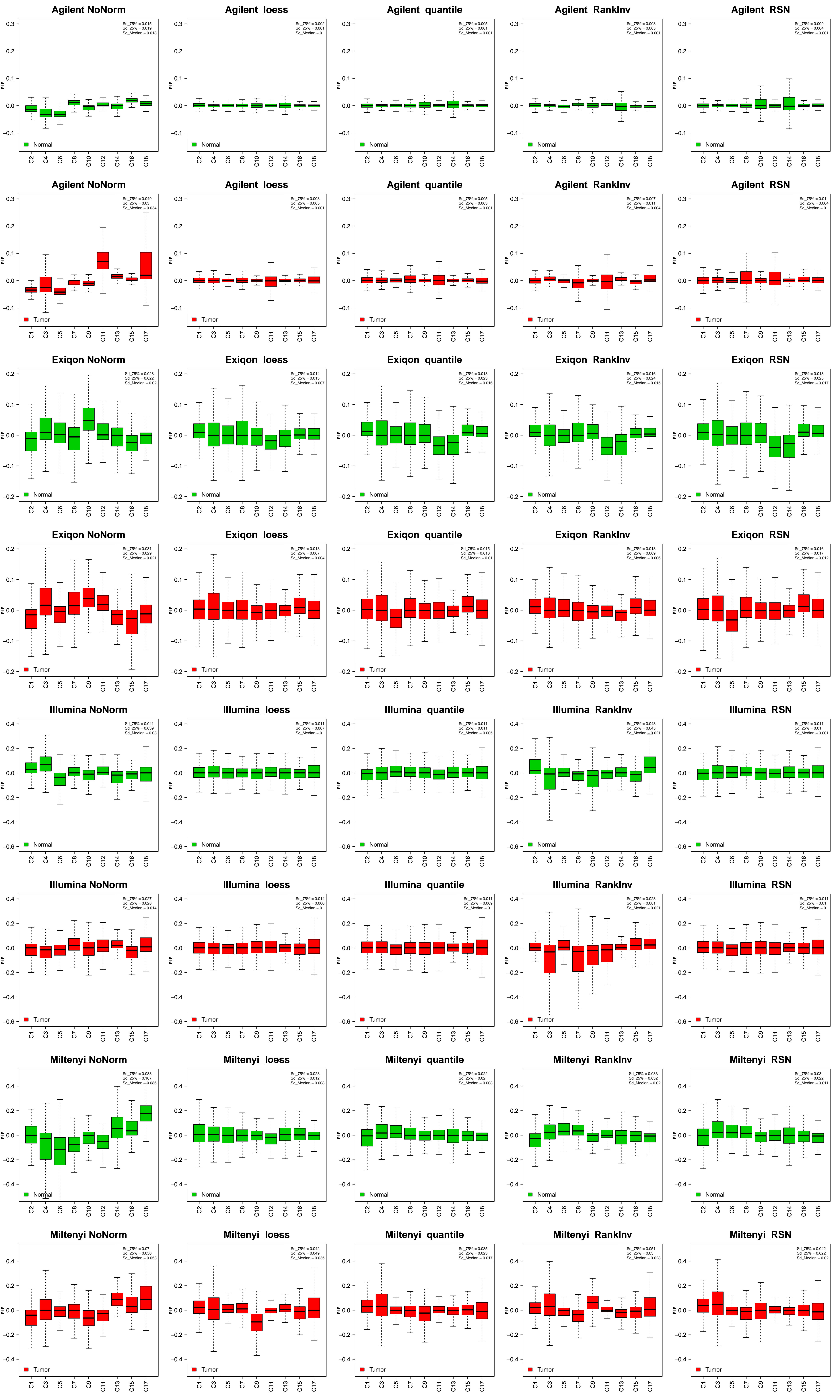

Supplement: Figure S2 — RLE plots. For each platform, RLE plots were generated separately for normal (green) and tumor (red) samples before and after normalization with one of the four methods taken in account. To evaluate the similarity of RLE values distribution we compared the standard deviations of the median, 25- and 75-percentile. (PDF) [file pone.0045105.s002.pdf]

Figure S3

Agilent

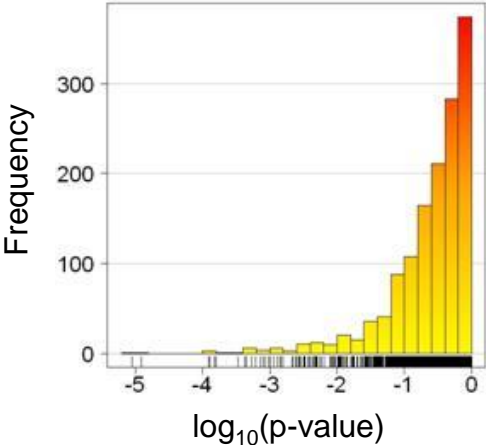

Exiqon

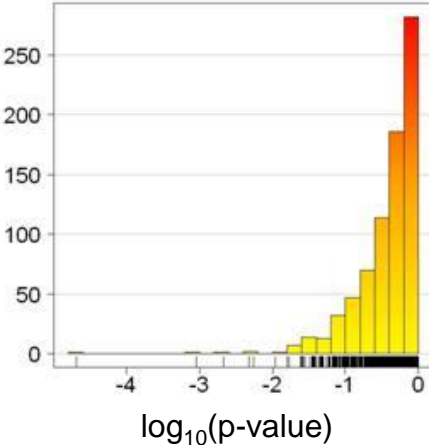

Illumina

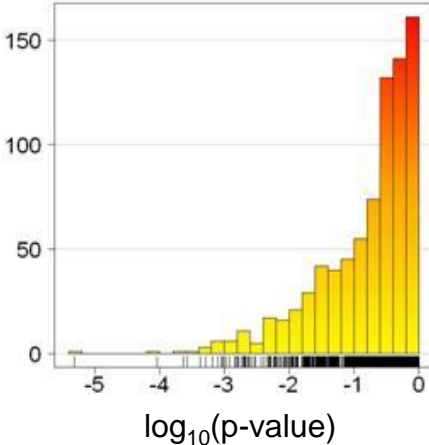

Miltenyi

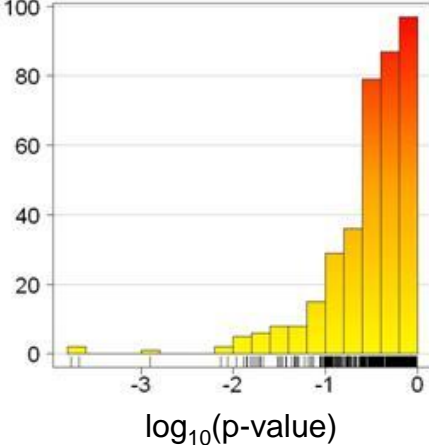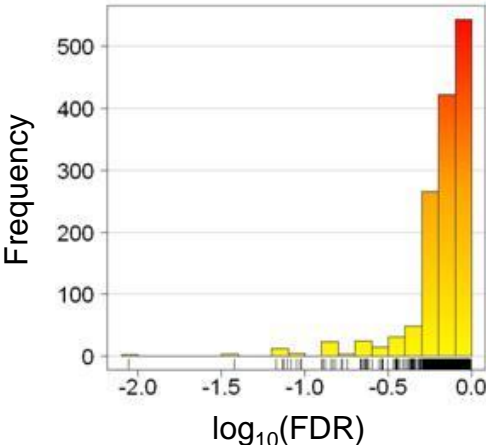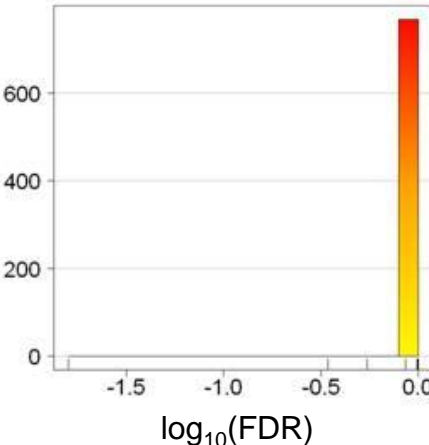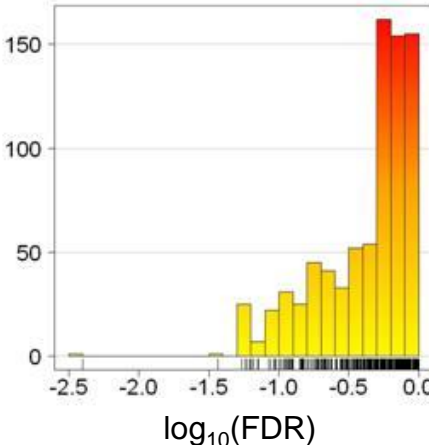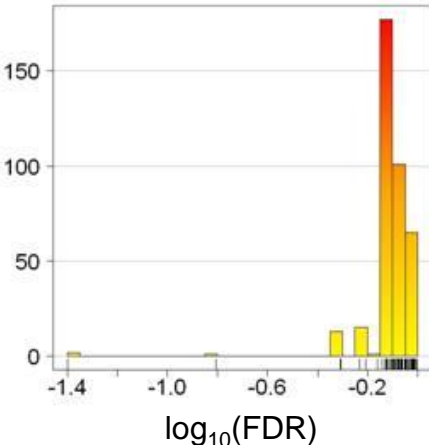

Supplement: Figure S3 — Comparison of differentially expressed miRNAs dependent on platform used. The histograms of log P-value and FDR frequency of the differentially expressed miRNAs in tumor/normal class comparisons are reported. (PDF) [file pone.0045105.s003.pdf]

**Figure S4**

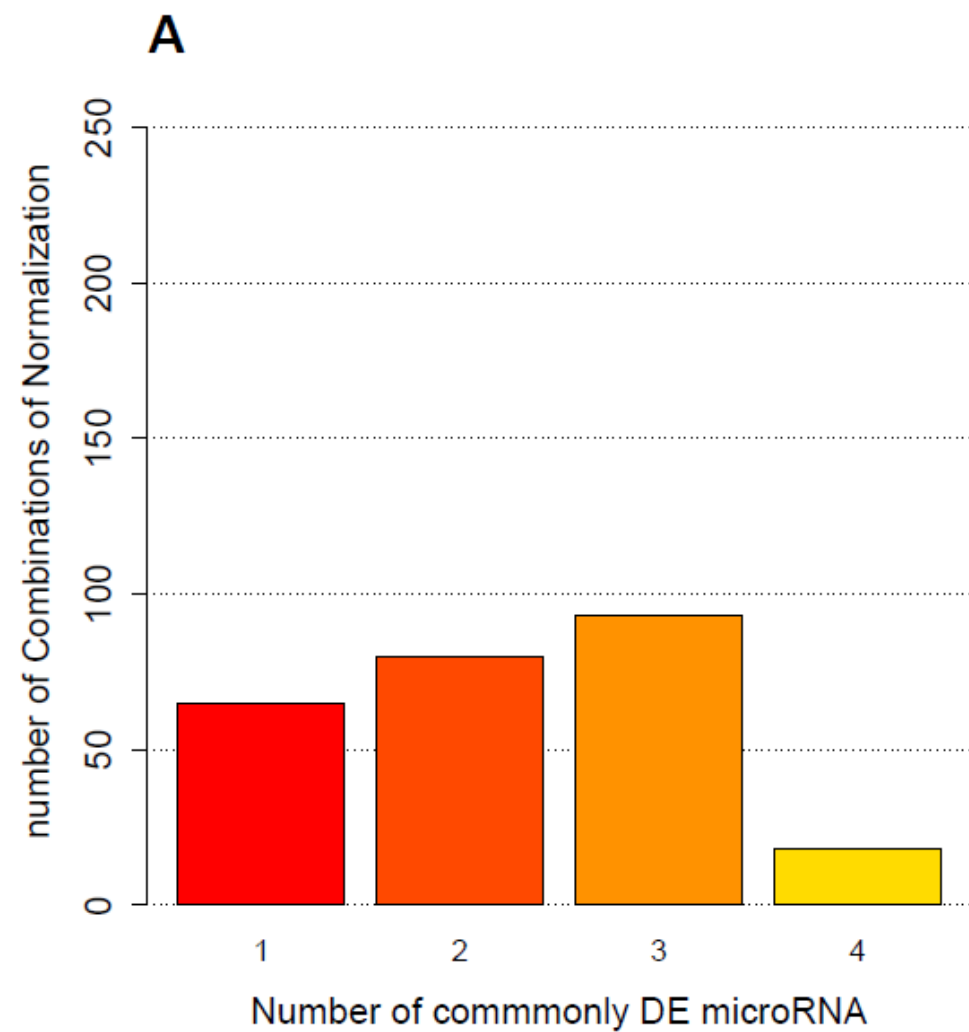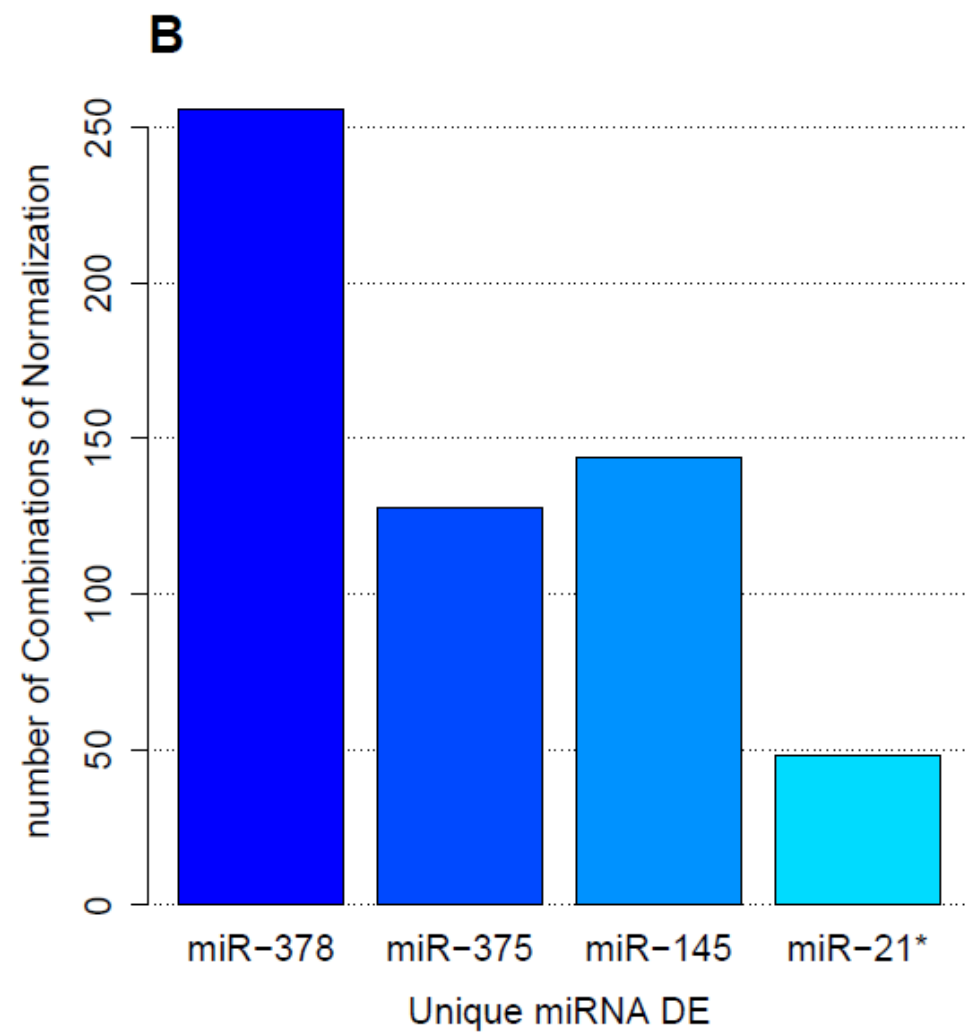

Supplement: Figure S4 — Impact of normalization on differential analysis. The four platforms were normalized using four different methods (Loess, Quantile, Rank Invariant, RSN). For each of the 256 possible combinations, the number of commonly differentially expressed miRNAs was computed and reported in (A). For microRNAs commonly detected as DE in at least one of the 256 combinations, the number of times they were selected is plotted in (B). (PDF) [file pone.0045105.s004.pdf]

Figure S5

A

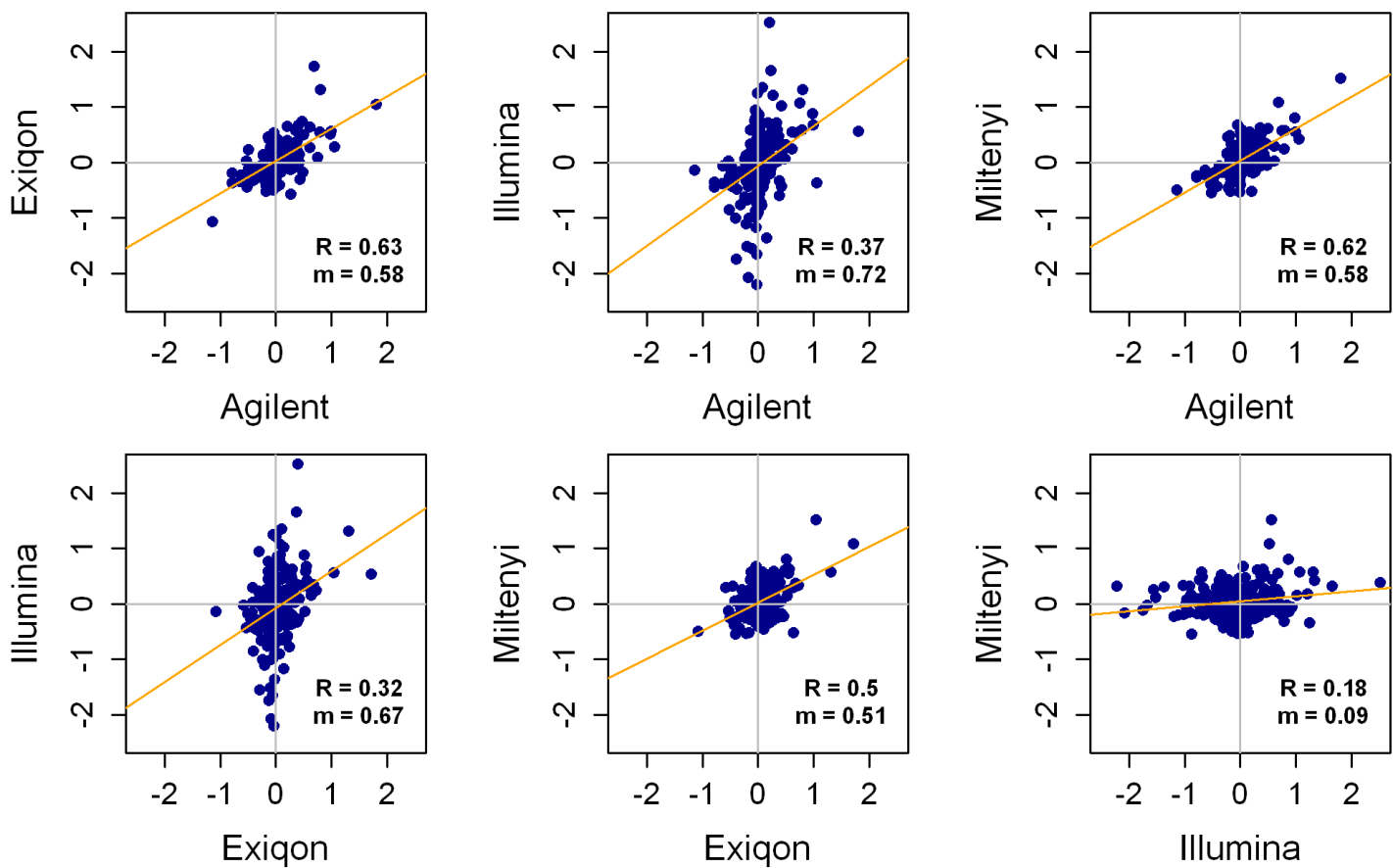

B

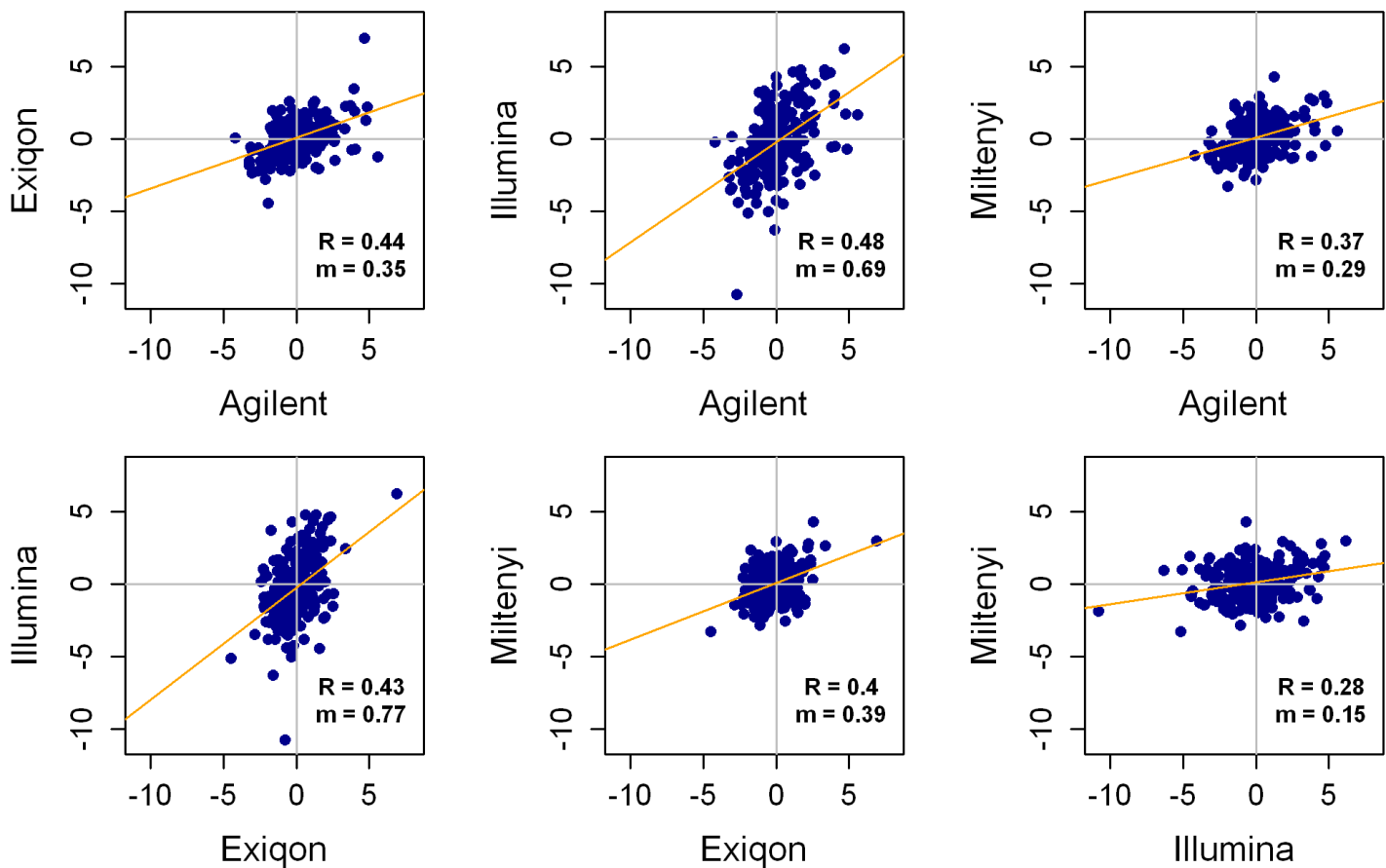

Supplement: Figure S5 — Common miRNA correlation. Pairwise correlation of log2 fold changes (A) and t-values (B) of the 233 miRNAs commonly detected by all platforms. Pearson correlation (R) and the slope (m) estimated by linear regression are shown. (PDF) [file pone.0045105.s005.pdf]

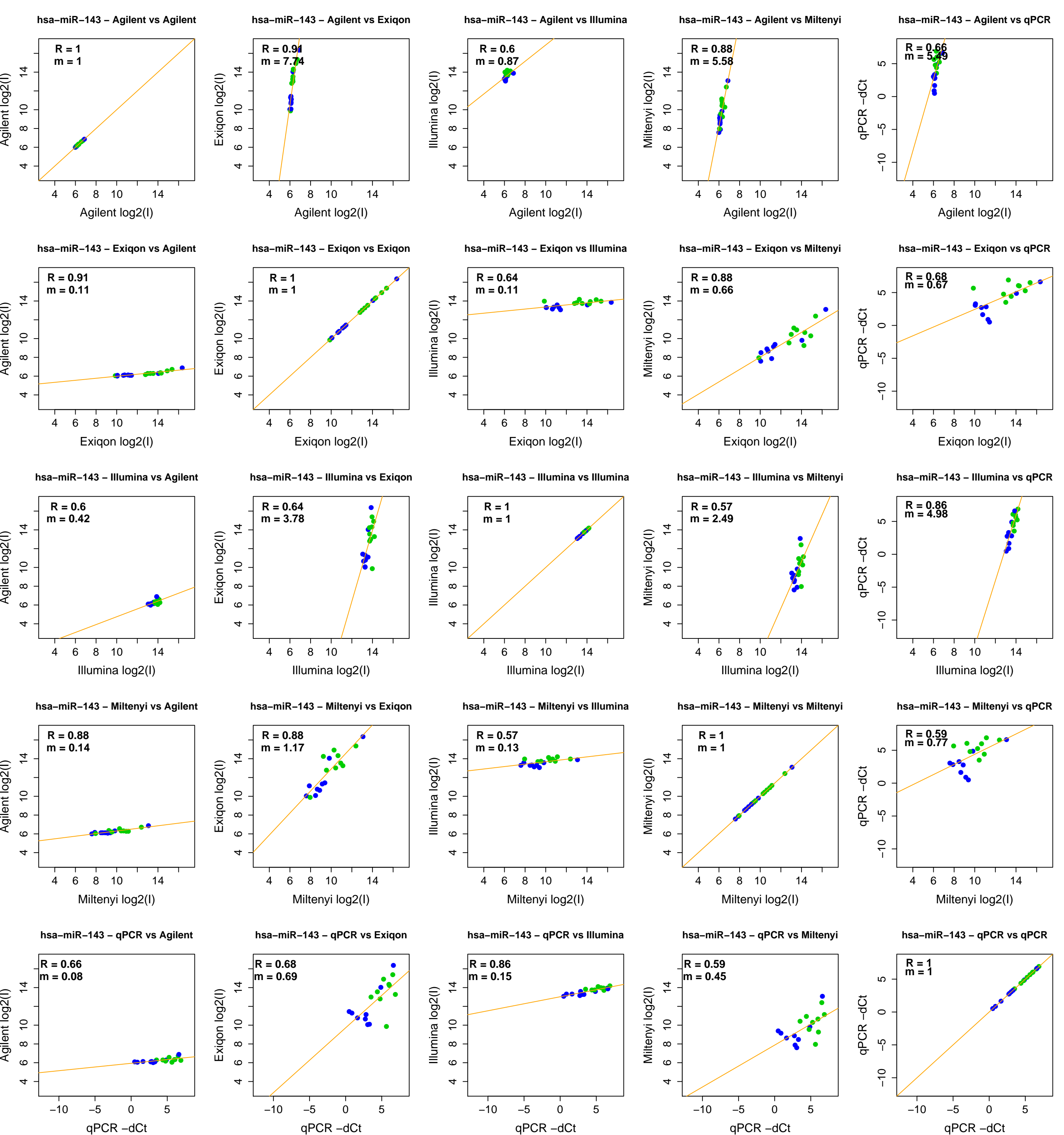

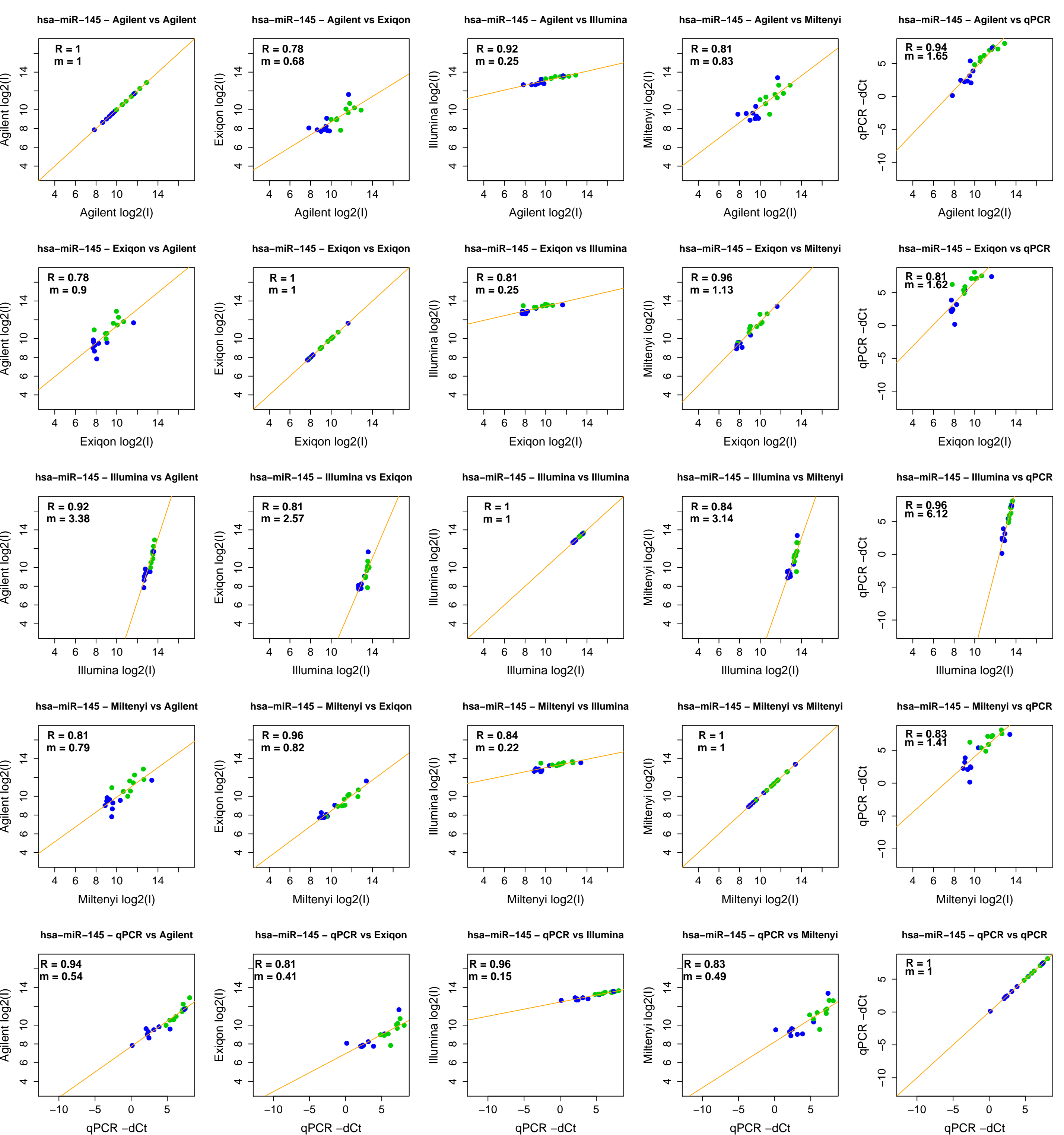

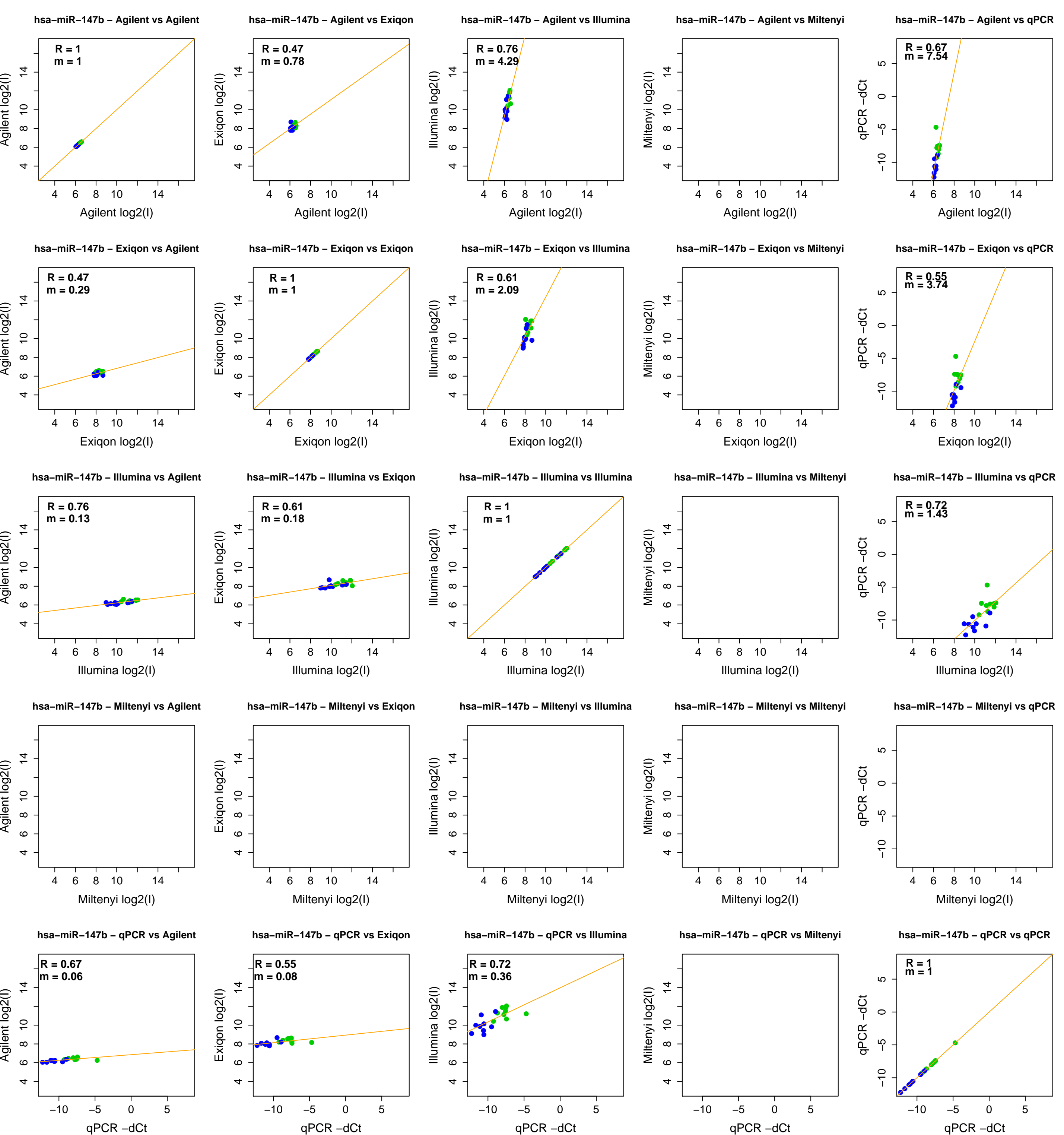

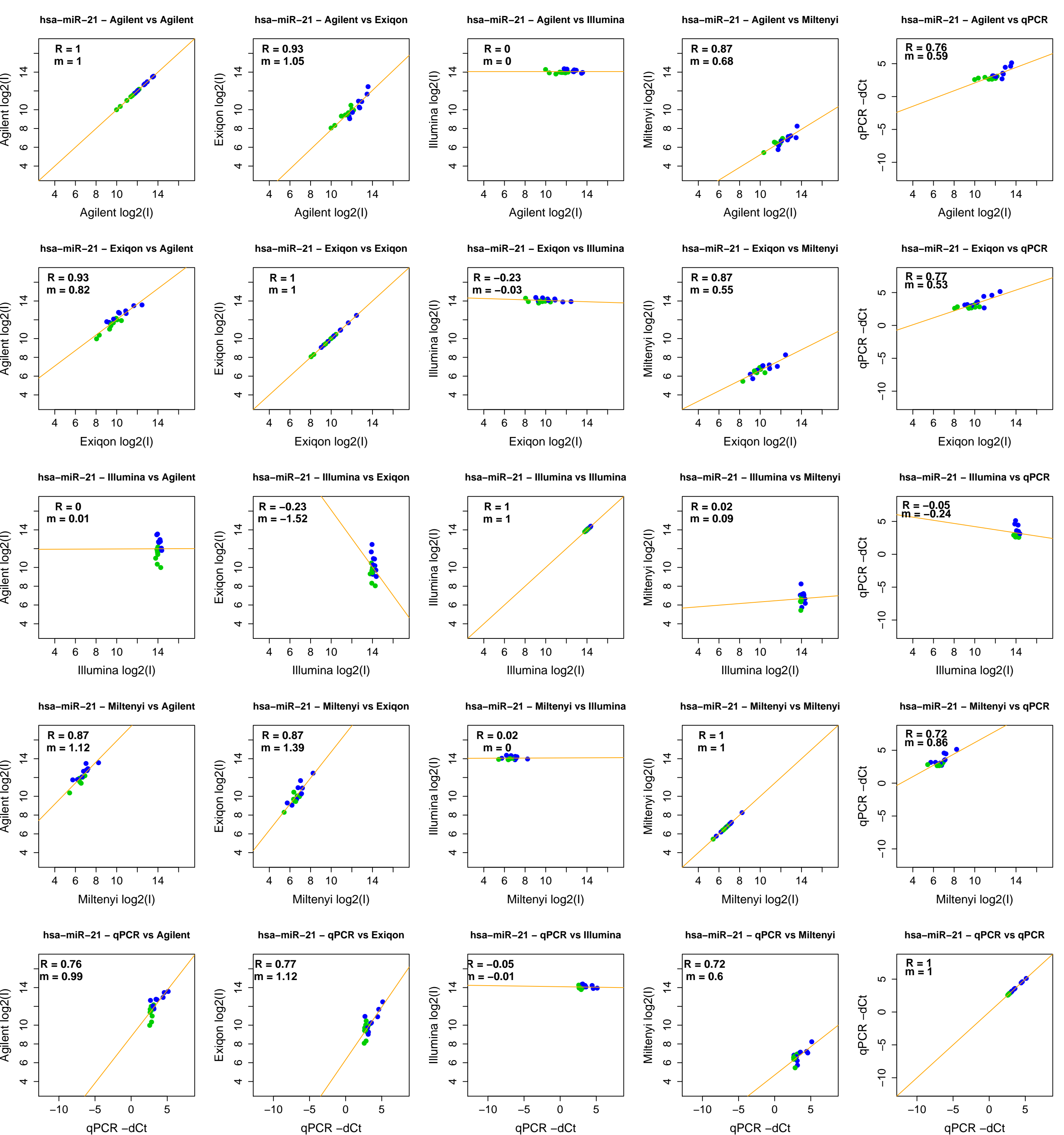

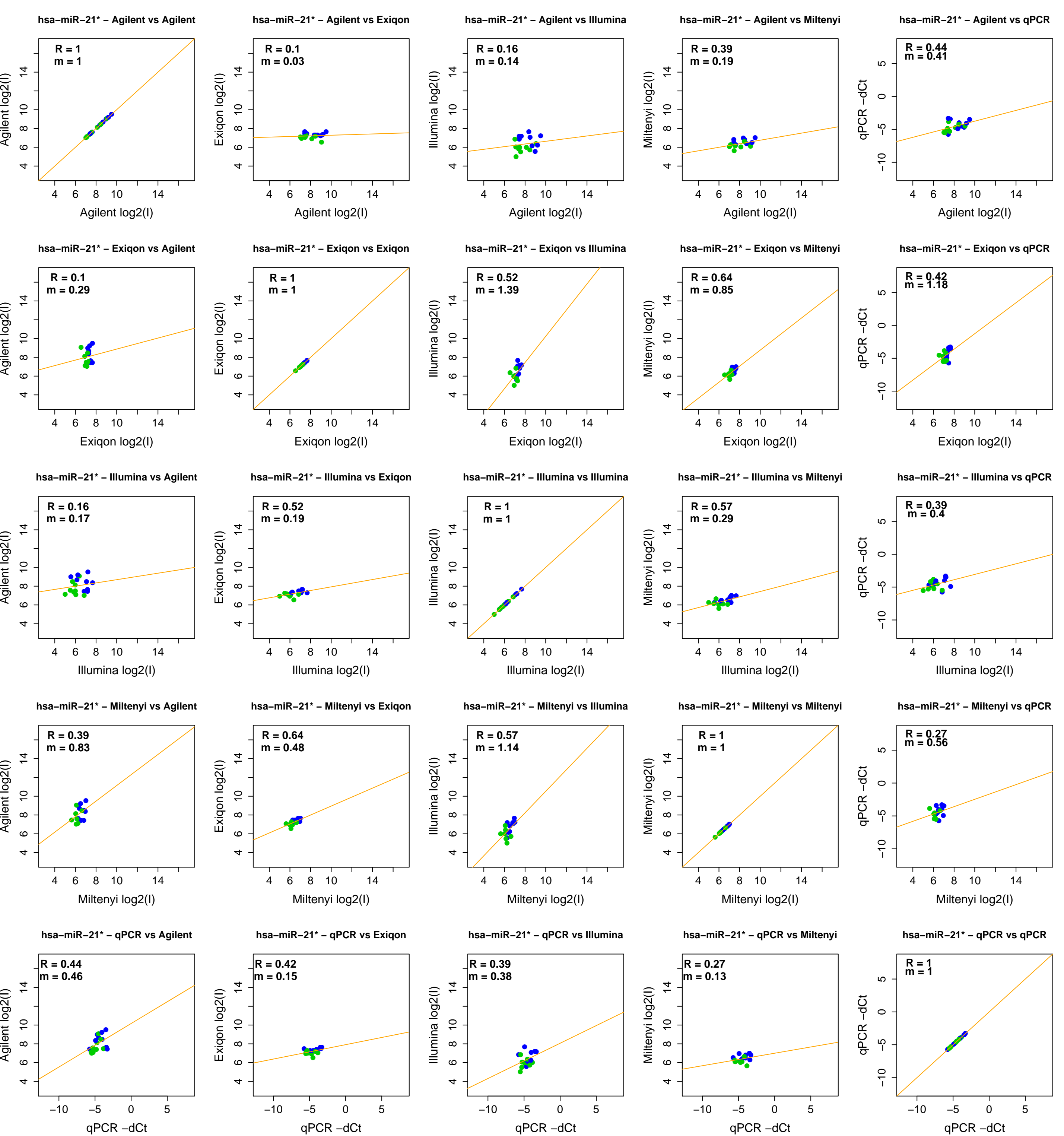

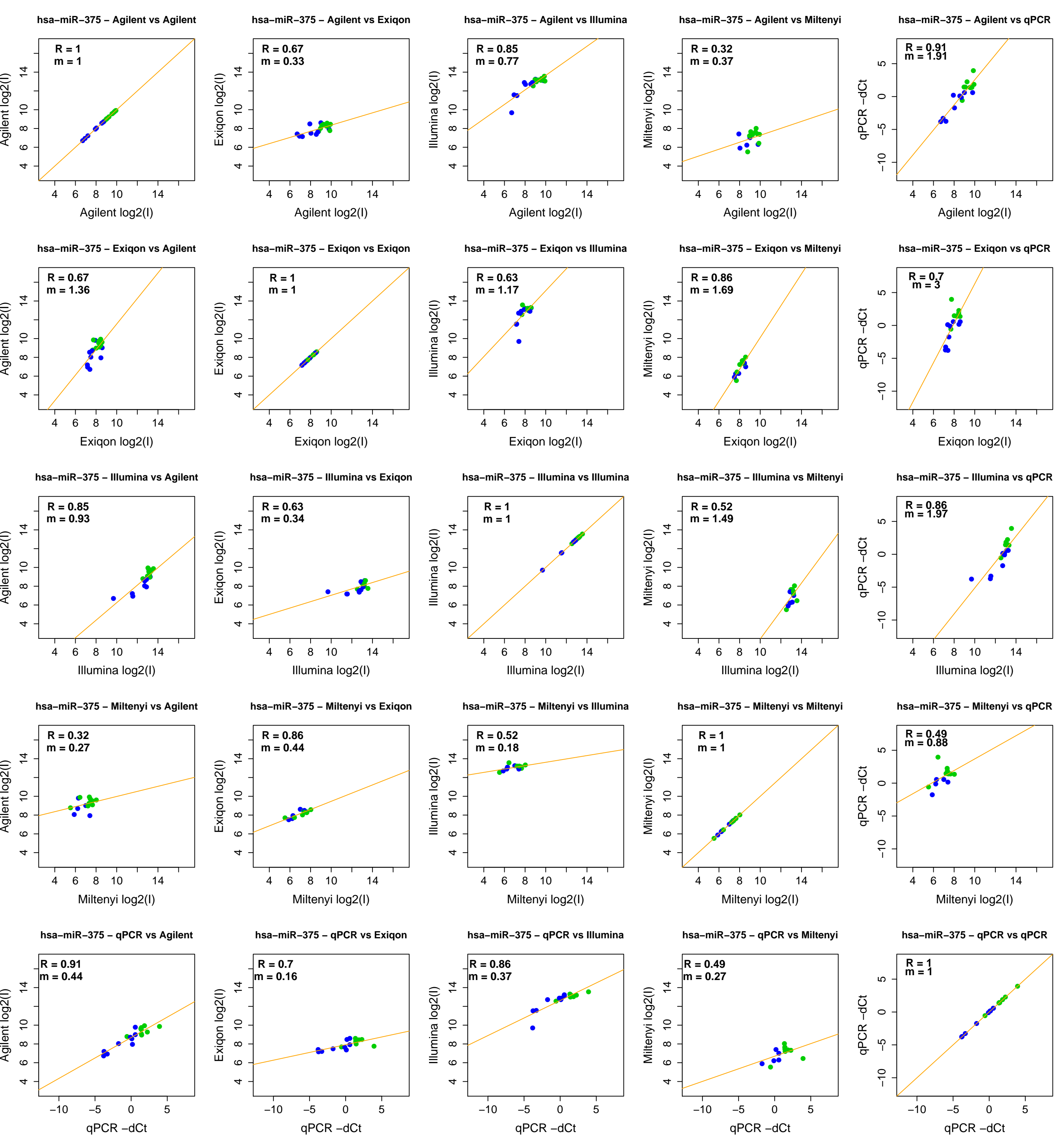

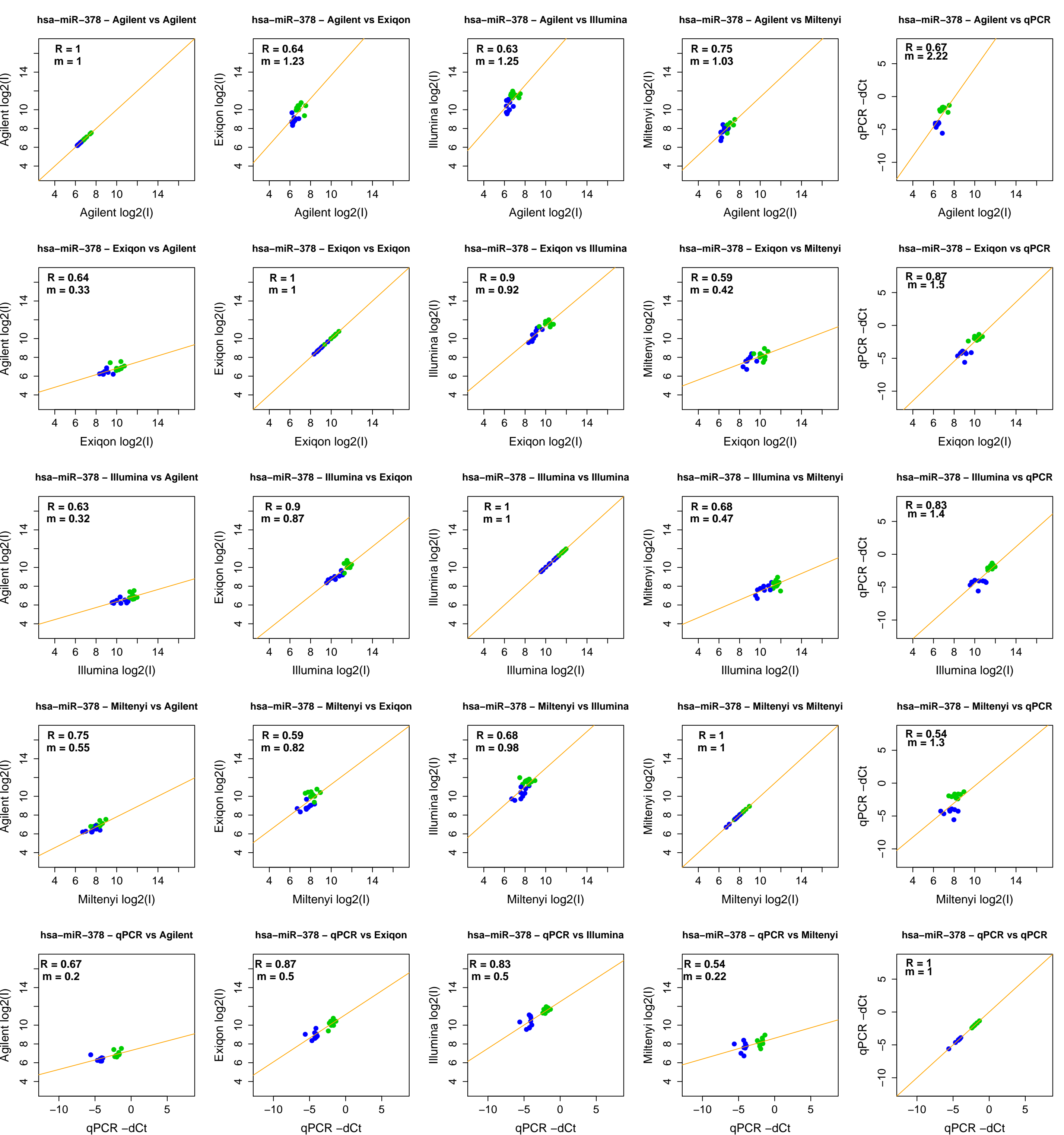

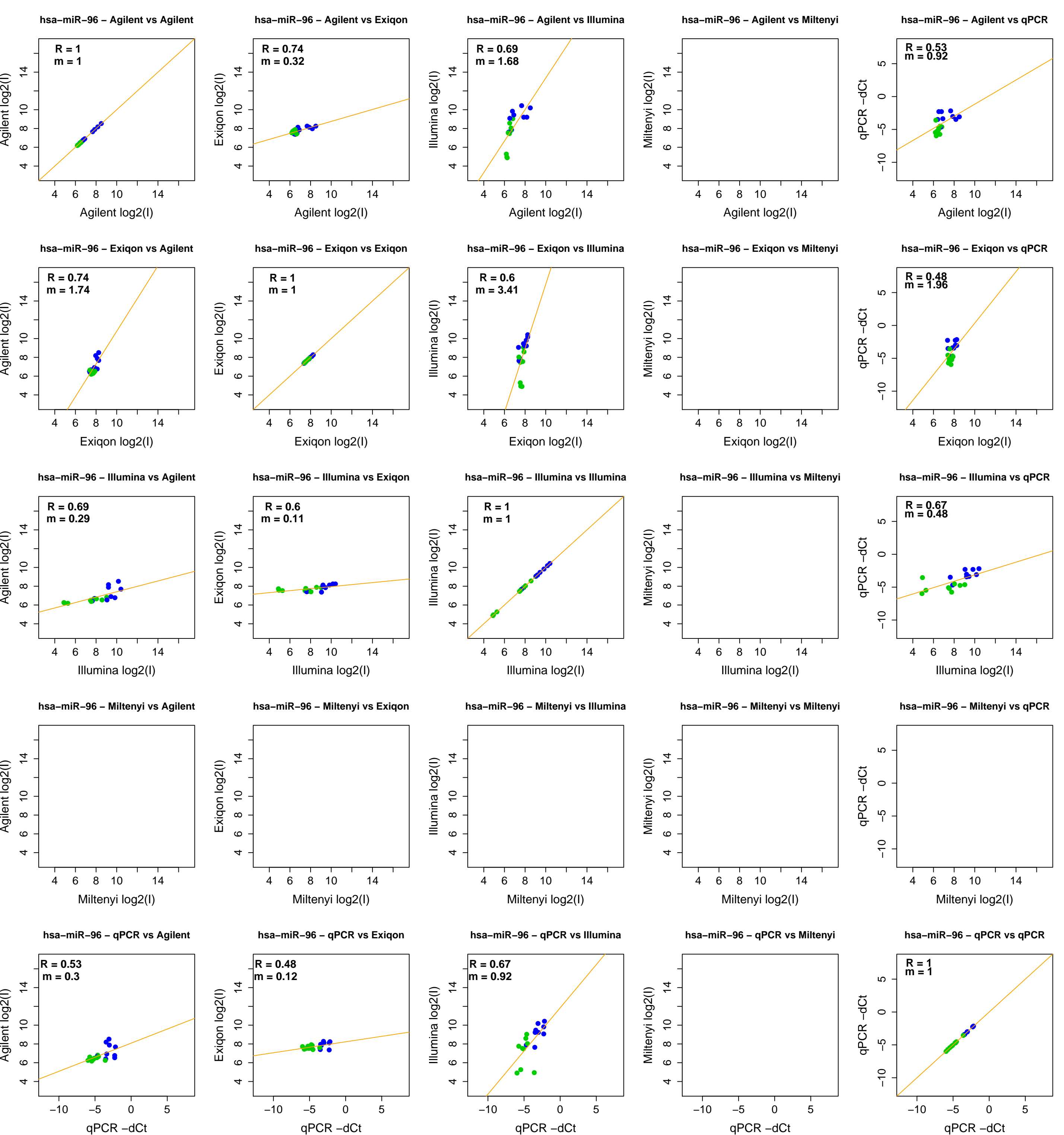

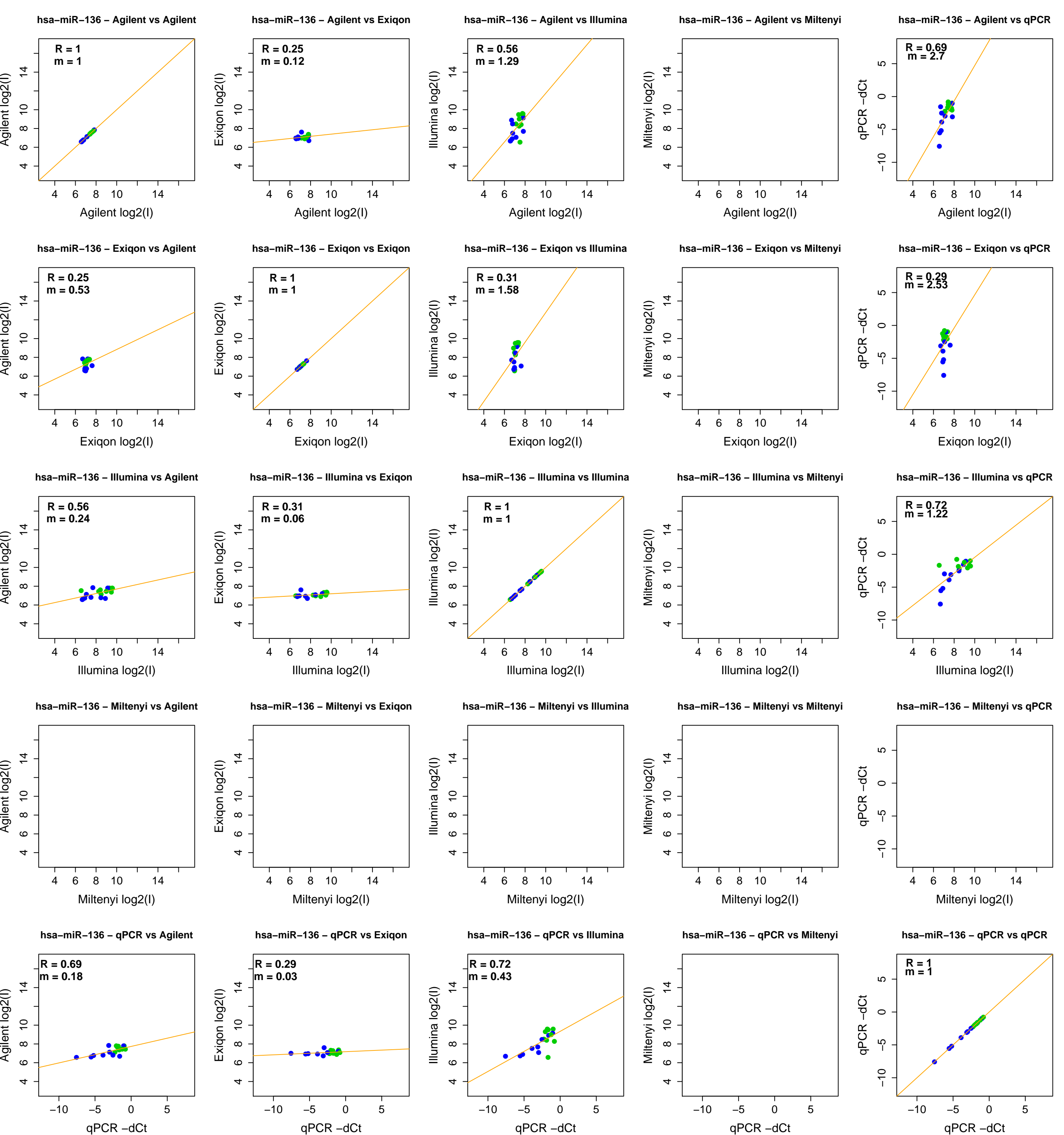

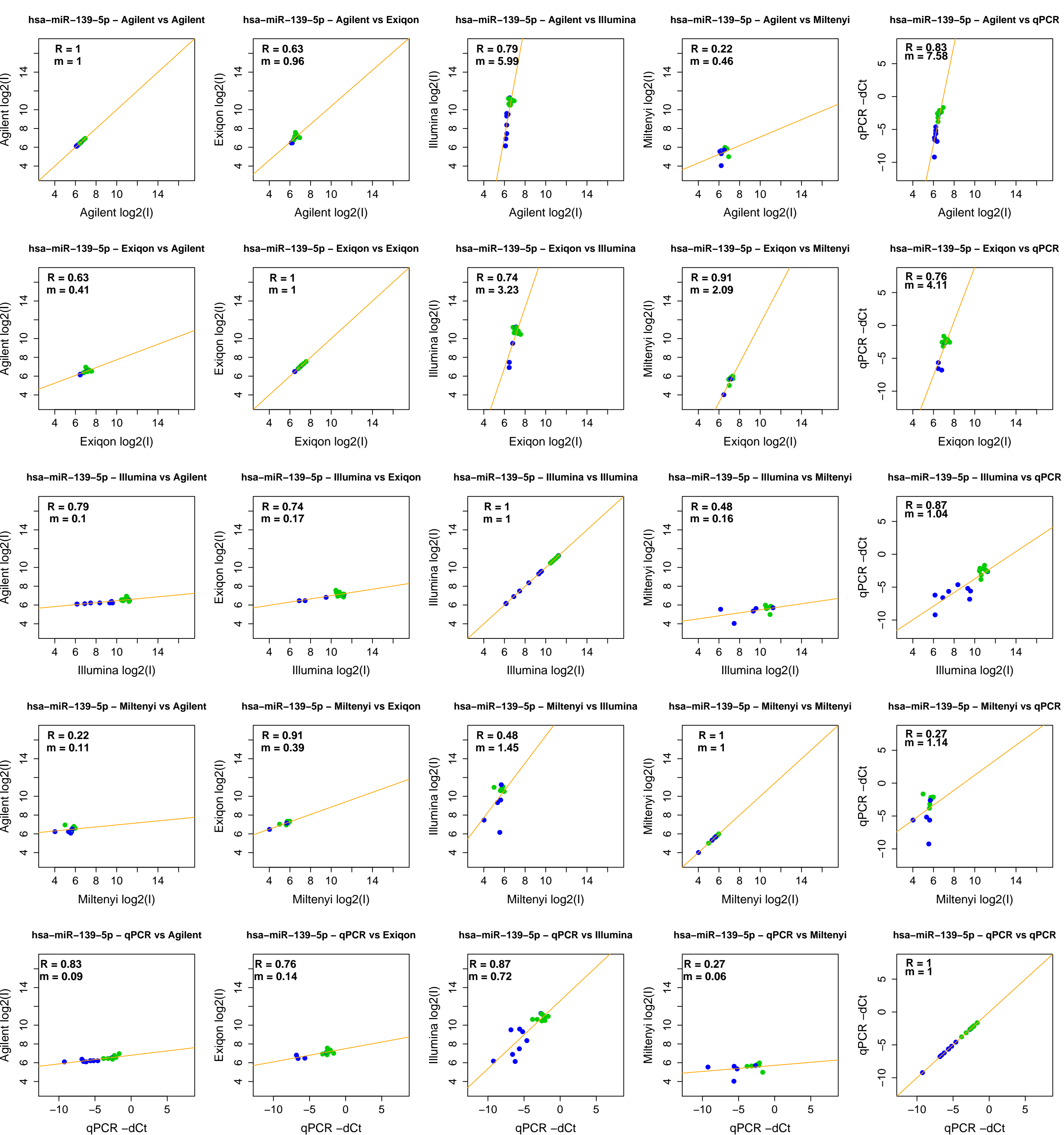

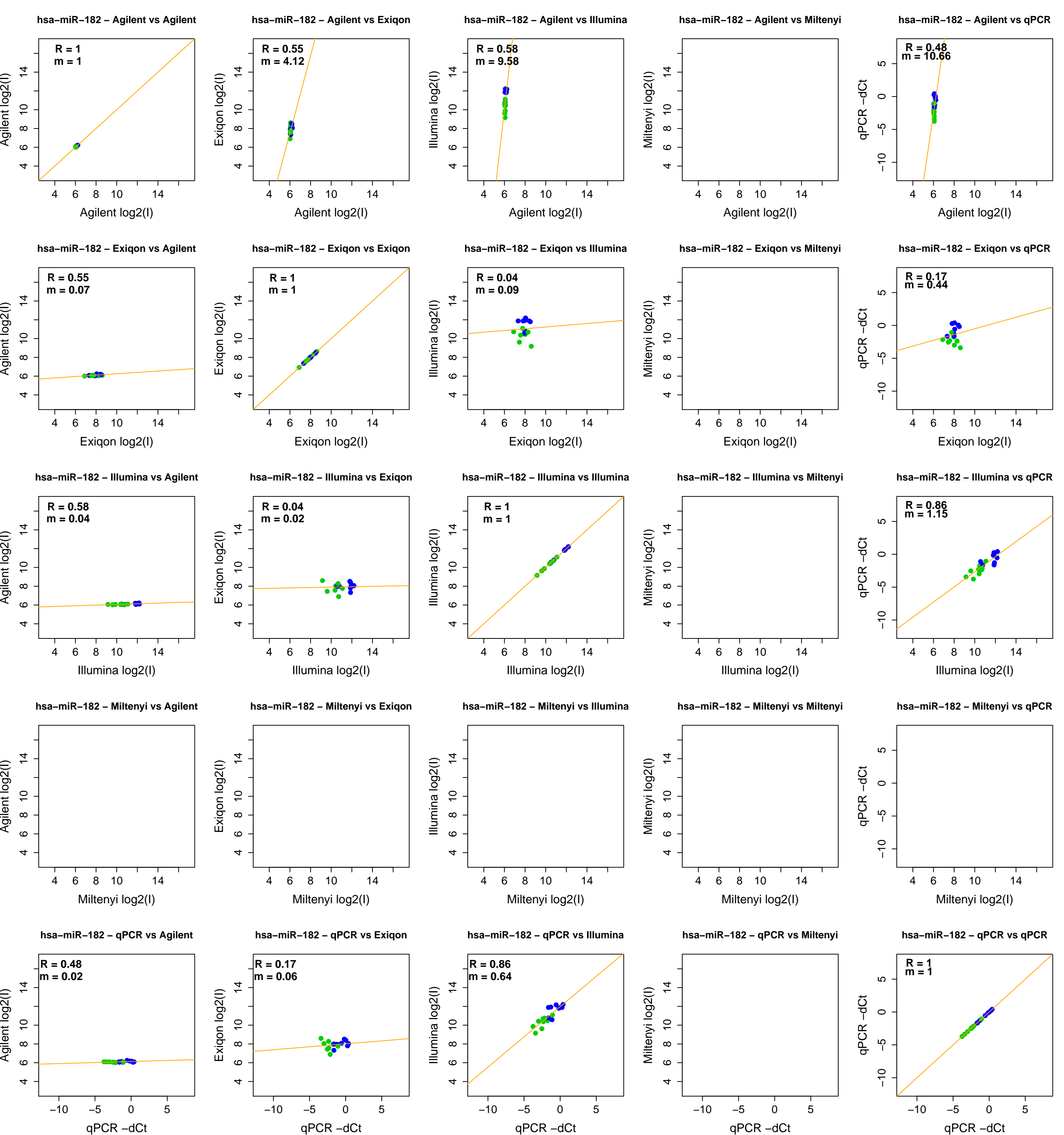

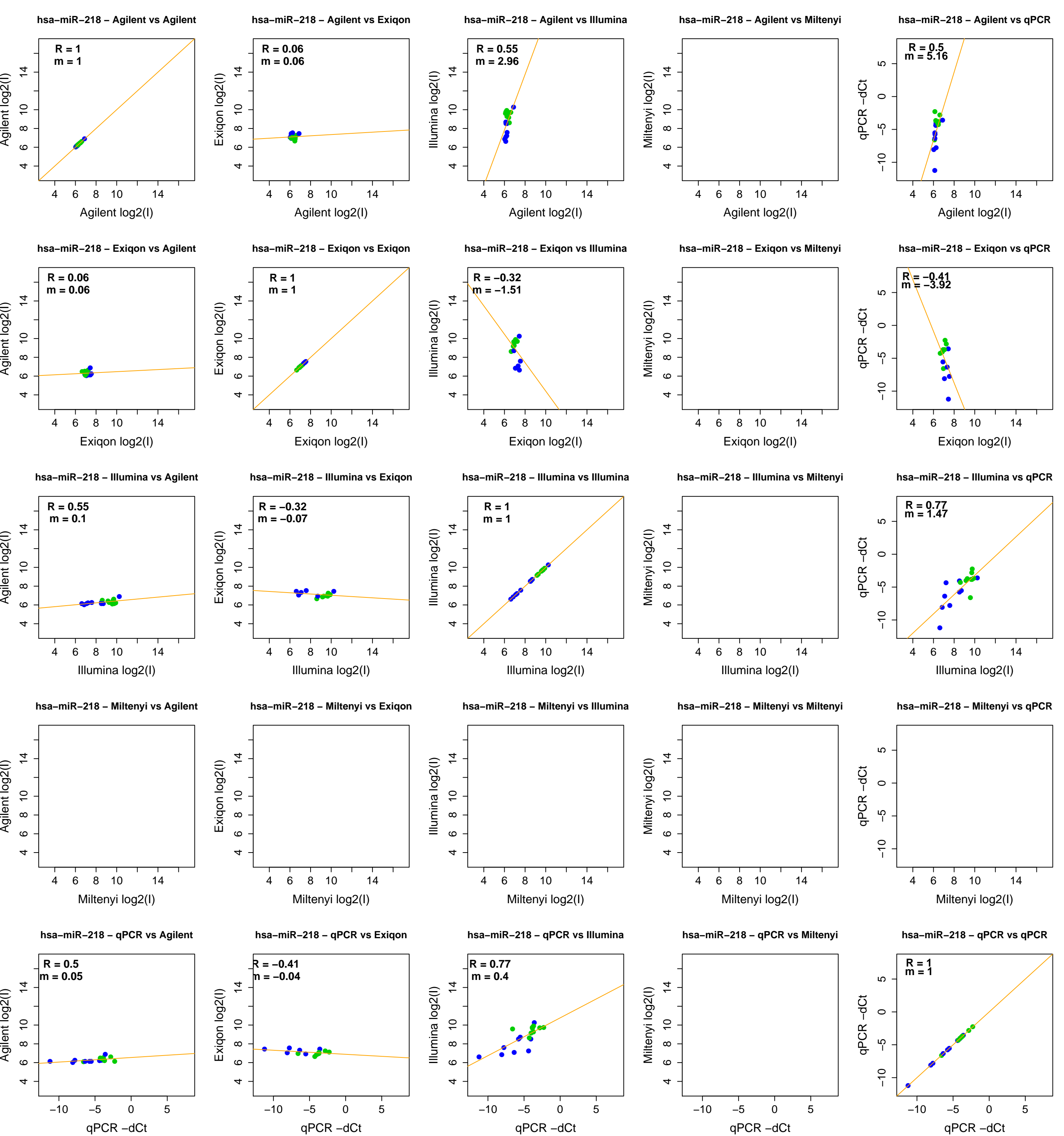

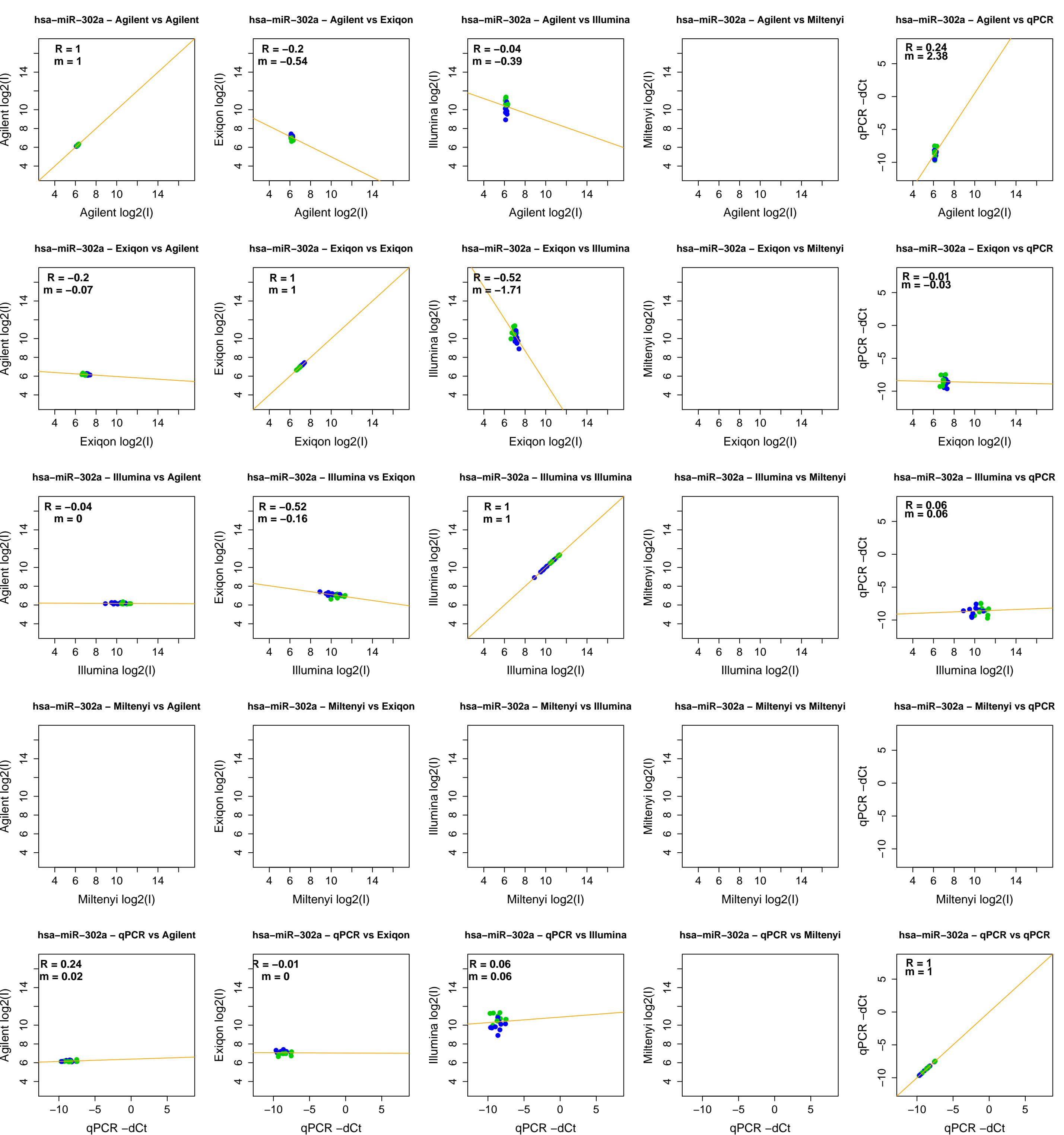

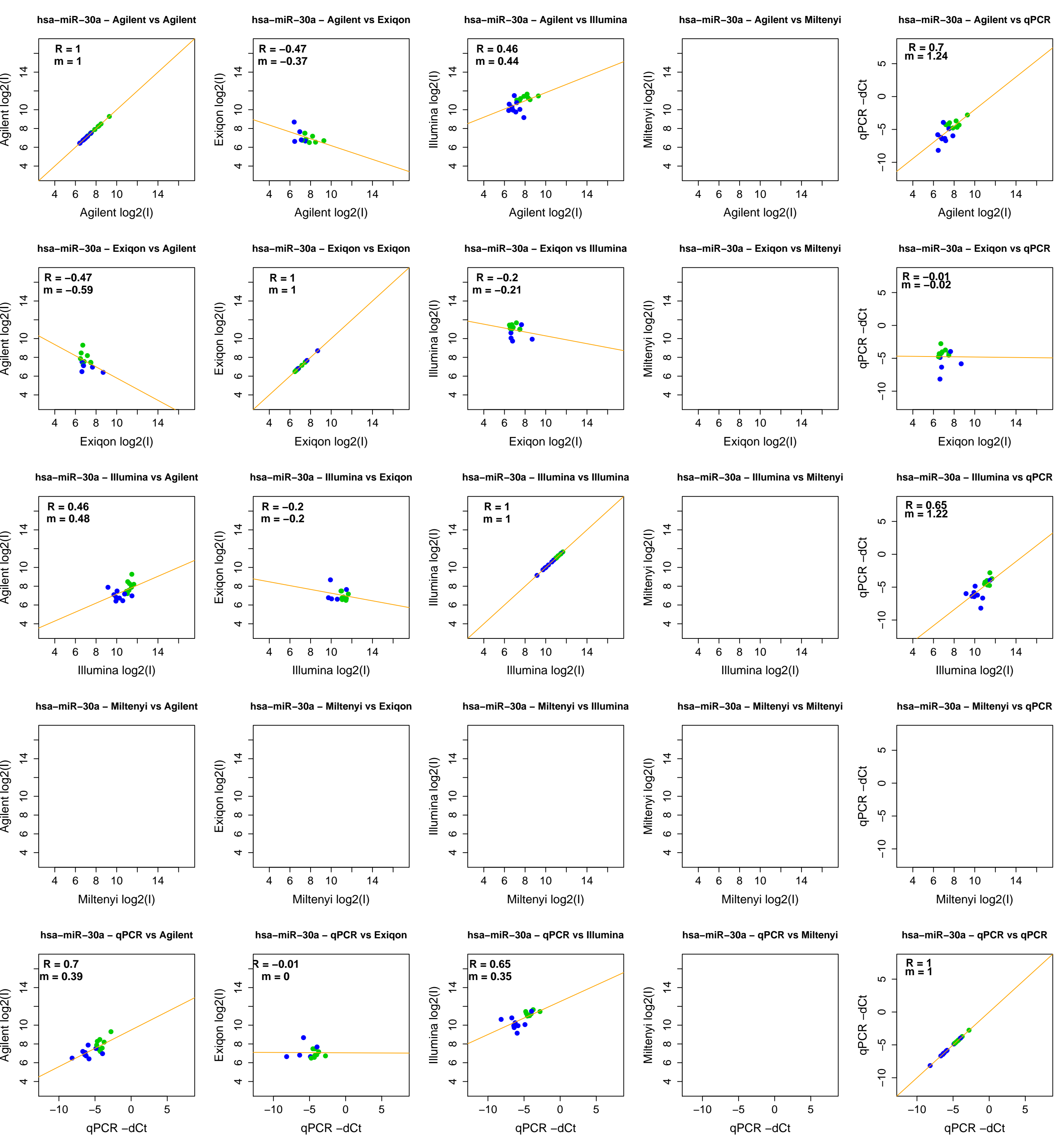

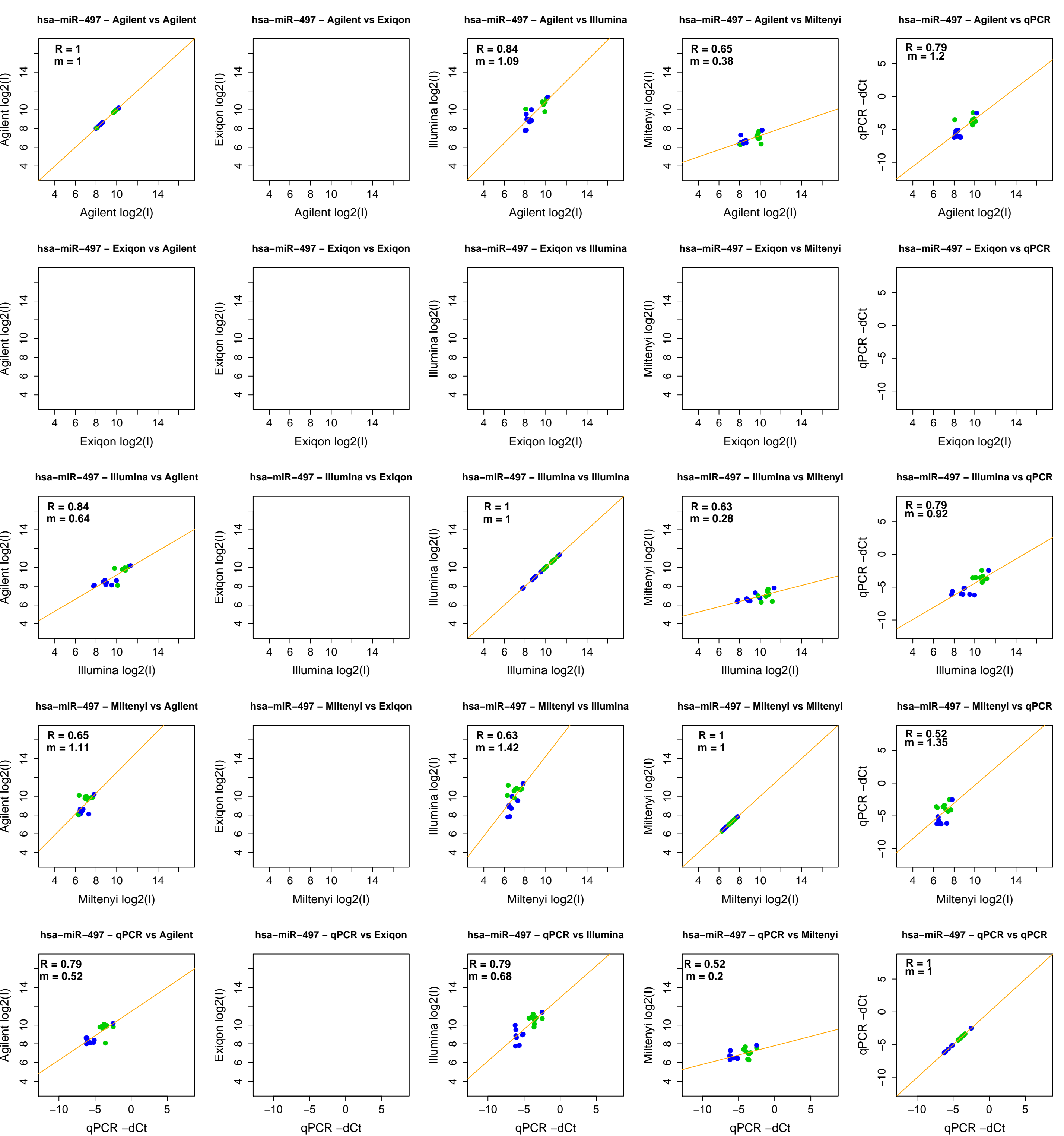

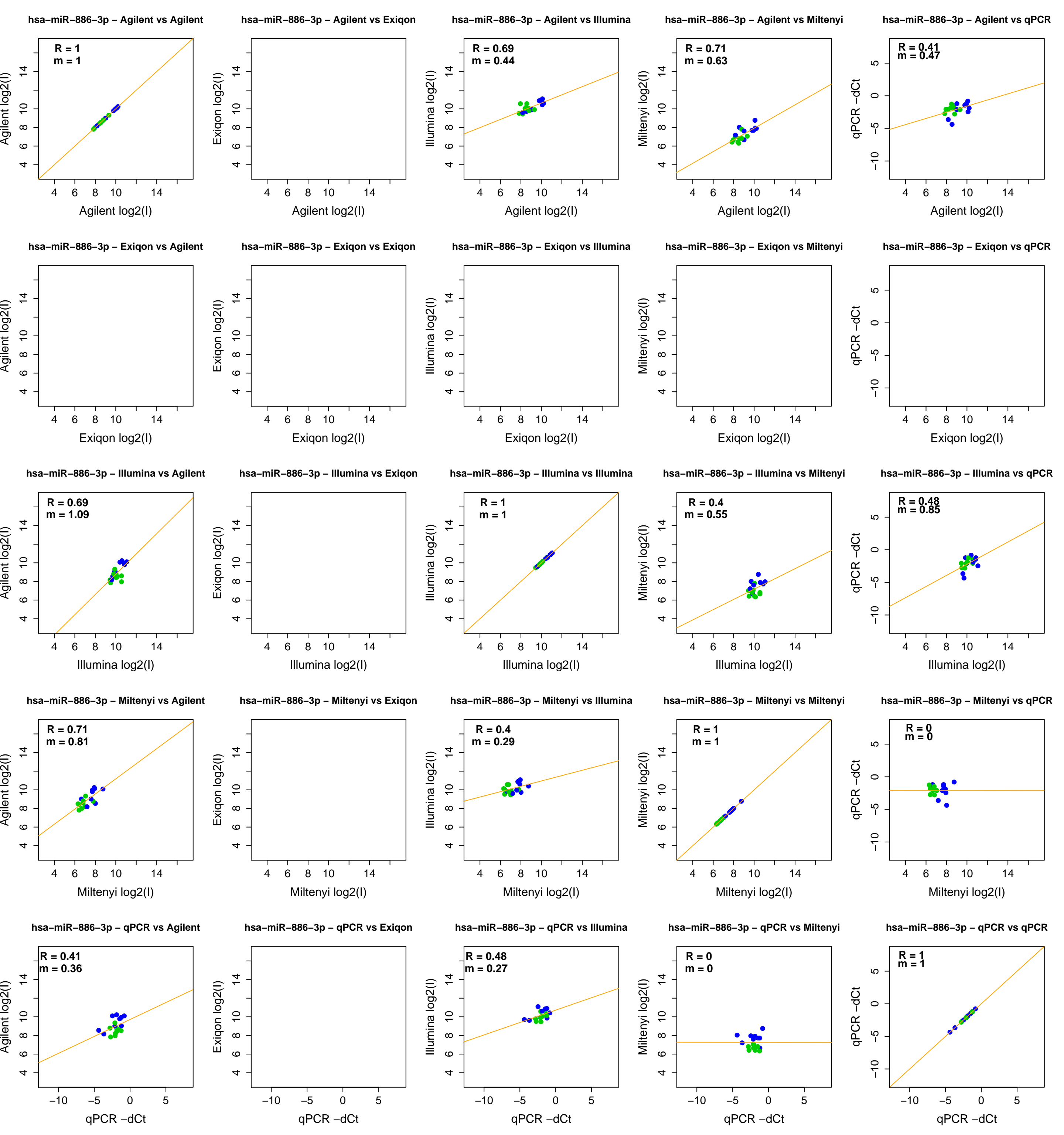

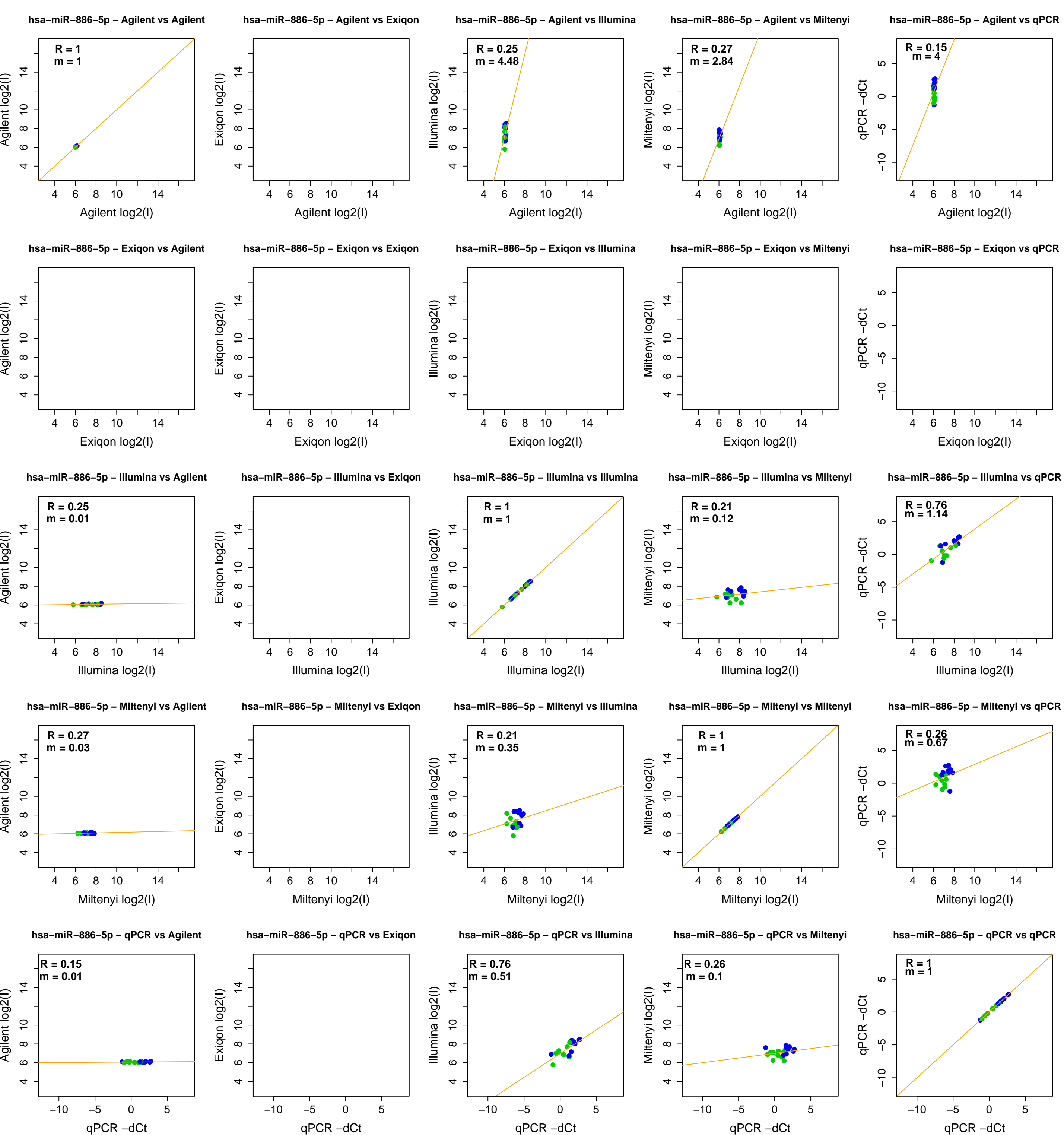

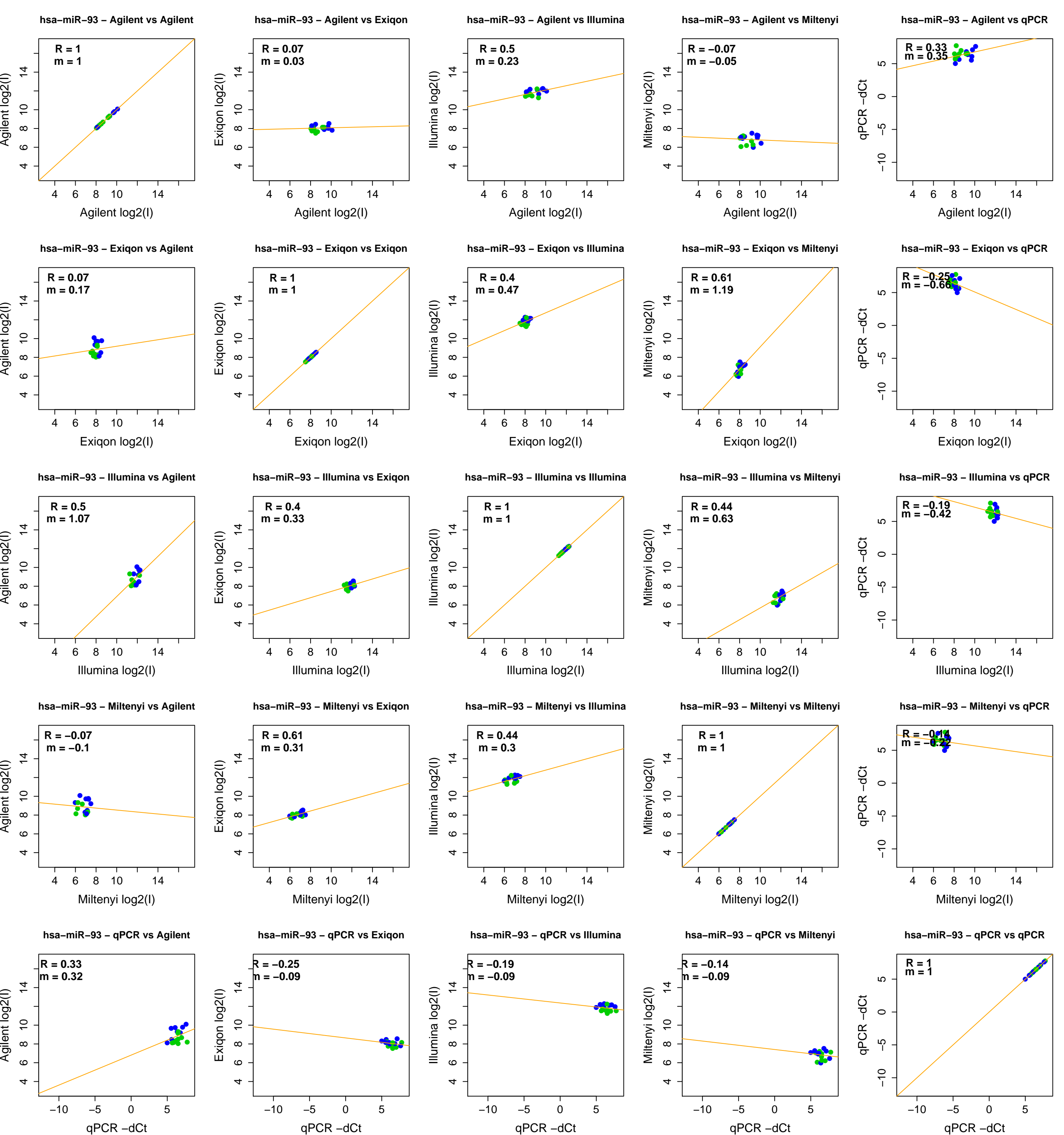

Supplement: File S1 — Pearson correlations between arrays and qPCR data for 18 selected miRNAs. (PDF) [file pone.0045105.s012.pdf]
