# Supplementary material for: Comparison of Microarray Platforms for Measuring Differential MicroRNA Expression in Paired Normal/Cancer Colon Tissues
Source: PLoS One. 2012 Sep 13;7(9):e45105. doi: 10.1371/journal.pone.0045105 (PMC3441572; doi:10.1371/journal.pone.0045105)
Supplement: Figure S6 — Batch effect correction. Moderated F-test (LIMMA package) was performed among classes defined by batches and F value distributions are plotted before and after applying the ComBat method [32] to both Agilent and Myltenyi expression data. The F threshold corresponding to a P<0.01 is plotted. After the correction, the number of miRNAs with a significant F values was reduced. (PDF) [file pone.0045105.s006.pdf]

Figure S6

A

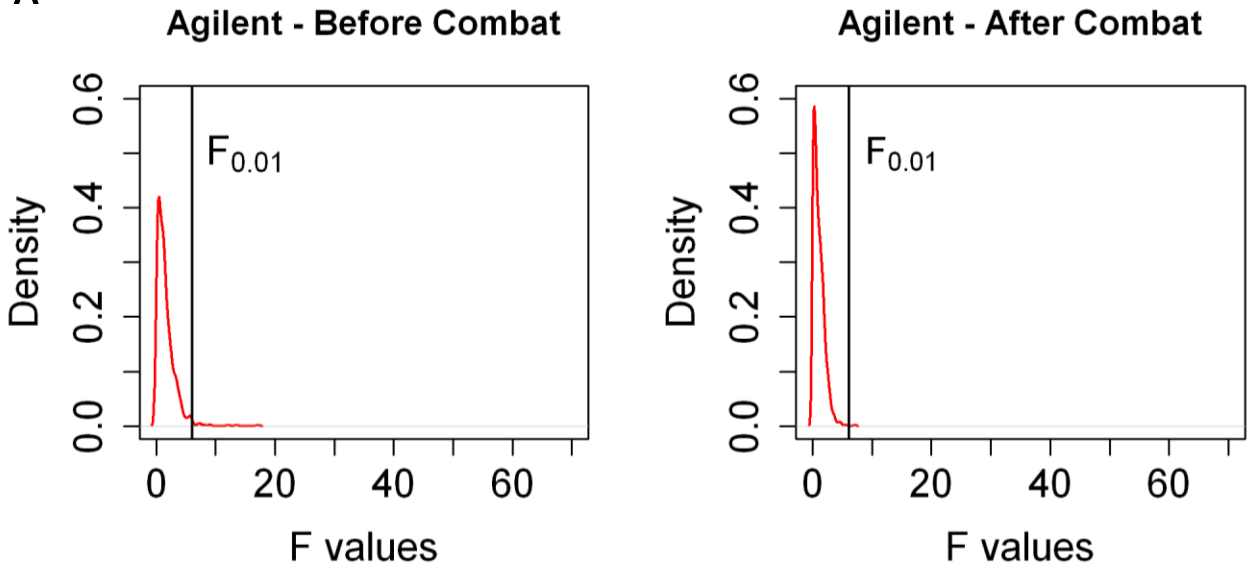
Agilent - Before Combat
Agilent - After Combat
Density
0.0
0.2
0.4
0.6
0
20
40
60
F values
$F_{0.01}$

B

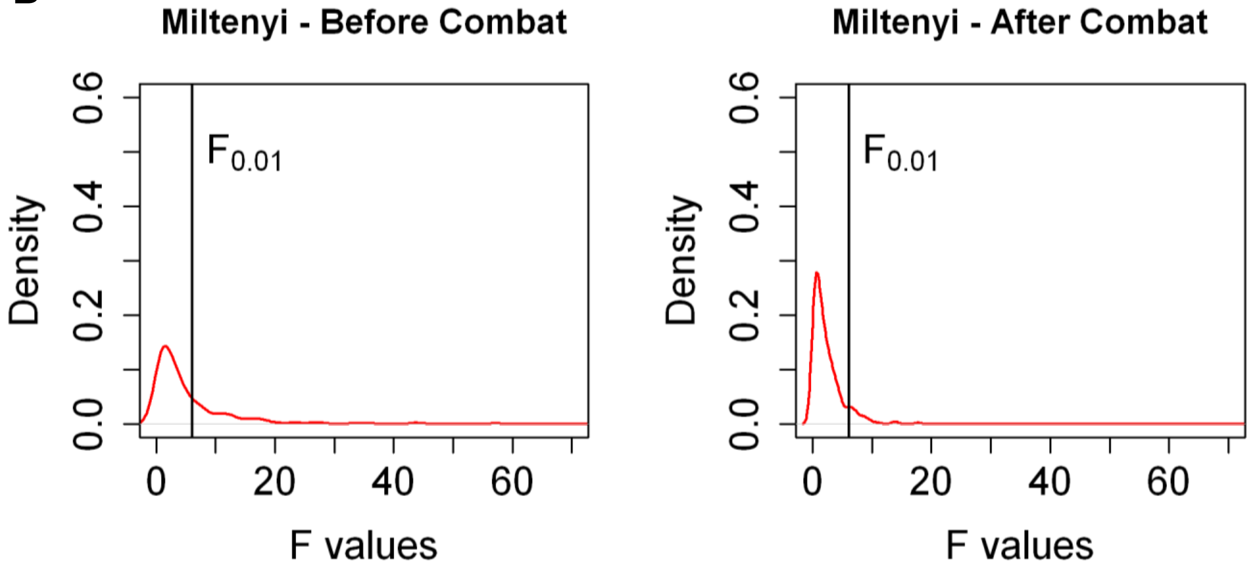
Miltenyi - Before Combat
Miltenyi - After Combat
Density
0.0
0.2
0.4
0.6
0
20
40
60
F values
$F_{0.01}$
