# Supplementary material for: Comparison of Microarray Platforms for Measuring Differential MicroRNA Expression in Paired Normal/Cancer Colon Tissues
Source: PLoS One. 2012 Sep 13;7(9):e45105. doi: 10.1371/journal.pone.0045105 (PMC3441572; doi:10.1371/journal.pone.0045105)
Supplement: Table S1 — Summary of cross-platform studies comparing more than three different platforms. (DOCX) [file pone.0045105.s007.docx]

**TABLE S1.** Summary of cross-platform studies comparing more than three different platforms

| **First author** |  | **Sato, F.** | **Pradervand, S.** | **Yauk, C.L.** | **Sah, S** | **Git, A.** | **Dreher, A.** |
| --- | --- | --- | --- | --- | --- | --- | --- |
| **Journal (year)** |  | PLoS ONE (2009) | BioTechniques (2010) | BMC Genomics (2010) | BMC Research Notes (2010) | RNA (2010) | Biochem Biophys Res Commun (2010) |
| **Platform** | **Agilent** | X | X | X | X | X |  |
|  | **Affymeterix** |  | X |  |  |  | X |
|  | **Ambion** | X |  |  | X | X |  |
|  | **Combimatrix** |  |  |  |  | X |  |
|  | **Exiqon** | X |  | X | X | X | X |
|  | **Illumina** |  | X |  | X | X |  |
|  | **Invitrogen** | X |  | X |  | X | X |
|  | **LCScience** |  |  | X |  |  |  |
|  | **Toray** | X |  |  |  |  |  |
| **Samples** |  | Liver/Prostate tissues | Heart/Brain normal tissues | Pool1(testicle, ovary, embrio)/ Pool2 (liver, heart, lung) | Spiked-in placental tissue | Pool normal breast tissues/ MCF7/ PMC42 | HPV-/HPV+ HaCat |
| **Normalization** |  | No Normalized | Quantile | Cyclic lowess | Quantile | dual channel: lowess single channel: quantile | lowess |
| **Origin** |  | Human | Human | Mouse | Human | Human | Human |
| **Replicates** |  | 3 | 4 | 2 | 7 | 4 | 3 |
| **Public access** |  | GEO: GSE13860 | NA | GEO: GSE19669 | GEO: GSE19248 | ArrayExpress: E-MTAB-96 | NA |
