# Supplementary material for: Comparison of Microarray Platforms for Measuring Differential MicroRNA Expression in Paired Normal/Cancer Colon Tissues
Source: PLoS One. 2012 Sep 13;7(9):e45105. doi: 10.1371/journal.pone.0045105 (PMC3441572; doi:10.1371/journal.pone.0045105)
Supplement: Table S2 — Clinical and pathological characteristics of patients. (DOCX) [file pone.0045105.s008.docx]

**TABLE S2.** Clinical and pathological characteristics of patients

| Patient | Age | Gender | Location | Grade | Stage | LN infiltration |
| --- | --- | --- | --- | --- | --- | --- |
| 1 | 73 | M | Rectosigmoid | G2 | pT2 | 0 |
| 2 | 32 | M | Rectosigmoid | G3 | pT3 | 13 |
| 3 | 55 | M | Rectosigmoid | G2 | pT4 | 2 |
| 4 | 43 | M | Sigmoid | G2 | pT4 | 2 |
| 5 | 35 | M | Colon | G2 | pT2 | 0 |
| 6 | 45 | F | Rectosigmoid | G2 | pT2 | 0 |
| 7 | 80 | F | Rectosigmoid | G2 | pT2 | 0 |
| 8 | 47 | M | Sigmoid | G2 | pT2 | 0 |
| 9 | 70 | M | Rectosigmoid | G3 | pT2 | 0 |
