# Supplementary material for: Comparison of Microarray Platforms for Measuring Differential MicroRNA Expression in Paired Normal/Cancer Colon Tissues
Source: PLoS One. 2012 Sep 13;7(9):e45105. doi: 10.1371/journal.pone.0045105 (PMC3441572; doi:10.1371/journal.pone.0045105)
Supplement: Table S3 — miRNA sets of DE miTNAs between colorectal cancer andnormal mucosa available from literature. (DOCX) [file pone.0045105.s009.docx]

| miRNA set | First author [Ref] | Description | Sample storage | Platform |
| --- | --- | --- | --- | --- |
| Arndt_tumor_vs_normal | Arndt GM [1] | DE miRNAs between 49 tumors and 4 normal mucosa (matched with 4 tumor) samples | frozen | Ambion |
| Sarver_ tumor _vs_norm | Sarver AL [2] | DE miRNAs between 80 tumors and 28 normal mucosa samples | frozen | Illumina |
| Piepoli_ tumor _vs_norm | Piepoli A [3] | DE miRNAs between 19 tumors and matched normal mucosa samples | frozen | Affimetrix |
| Mosakhani_ tumor _vs_norm | Mosakhani N [4] | DE miRNAs between 55 metastatic tumors and one commercial normal mucosa | FFPE^§^ | Agilent |
| Motoyama_ tumor _vs_norm | Motoyama K [5] | DE miRNAs between 4 tumors and matched normal mucosa samples | frozen | Agilent |

**Table S3**: miRNA sets of DE miRNAs between colorectal cancer and normal mucosa available from literature

§FFPE = formalin fixed paraffin embedded

Reference List

(1) Arndt GM, Dossey L, Cullen LM, Lai A, Druker R, et al. (2009) Characterization of global microRNA expression reveals oncogenic potential of miR-145 in metastatic colorectal cancer. BMC Cancer 9: 374.

(2) Sarver AL, French AJ, Borralho PM, Thayanithy V, Oberg AL, et al. (2009) Human colon cancer profiles show differential microRNA expression depending on mismatch repair status and are characteristic of undifferentiated proliferative states. BMC Cancer 9: 401.

(3) Piepoli A, Tavano F, Copetti M, Mazza T, Palumbo O, et al. (2012) Mirna expression profiles identify drivers in colorectal and pancreatic cancers. PLoS One 7: e33663.

(4) Mosakhani N, Sarhadi VK, Borze I, Karjalainen-Lindsberg ML, Sundstrom J, et al. (2012) MicroRNA profiling differentiates colorectal cancer according to KRAS status. Genes Chromosomes Cancer 51: 1-9.

(5) Motoyama K, Inoue H, Takatsuno Y, Tanaka F, Mimori K, et al. (2009) Over- and under-expressed microRNAs in human colorectal cancer. Int J Oncol 34: 1069-75.
