# Supplementary material for: Comparison of Microarray Platforms for Measuring Differential MicroRNA Expression in Paired Normal/Cancer Colon Tissues
Source: PLoS One. 2012 Sep 13;7(9):e45105. doi: 10.1371/journal.pone.0045105 (PMC3441572; doi:10.1371/journal.pone.0045105)
Supplement: Table S4 — miRNA chromosome location, relative expression and comparison with literature data. (DOCX) [file pone.0045105.s010.docx]

| **Table S4:** miRNA chromosome location, relative expression and comparison with literature | | | | | | |
| --- | --- | --- | --- | --- | --- | --- |
|  |  |  |  |  |  |  |
| **miR** | **Location** | **Agilent_fold (class T /class N )** | **Exiqon_fold (class T /class N )** | **Illumina_fold (class T /class N )** | **Miltenyi_fold (class T /class N )** | **Upregulated in:*** |
| hsa-miR-452* | Xq28 | ND | 1.05 | 1.32 | ND |  |
| hsa-miR-513b | Xq27.3 | 1.03 | 1.02 | 1.49 | 1.13 |  |
| hsa-miR-890 | Xq27.3 | ND | 0.97 | 1.56 | 0.86 |  |
| hsa-miR-421 | Xq13.2 | 1.02 | 1.00 | 1.67 | ND |  |
| hsa-miR-221 | Xp11.3 | 1.22 | 1.12 | 1.17 | 1.03 | T§ |
| hsa-miR-501-5p | Xp11.23 | 1.10 | 1.03 | 1.57 | 1.15 |  |
| hsa-miR-1184 | NA | 1.02 | 0.79 | 1.40 | ND |  |
| hsa-miR-1201 | NA | ND | NP | 1.78 | ND |  |
| hsa-miR-1202 | NA | 0.47 | 0.82 | 1.29 | 0.75 |  |
| hsa-miR-1286 | NA | 1.03 | 0.88 | 1.32 | 1.12 |  |
| hsa-miR-147 | 9q33.2 | ND | 1.05 | 0.25 | ND |  |
| hsa-miR-491-5p | 9p21.3 | 1.03 | 0.91 | 2.27 | 1.18 |  |
| hsa-miR-124 | 8p23.1 | 0.79 | 0.97 | 0.72 | 1.12 | N |
| hsa-miR-182 | 7q32.2 | 1.04 | 1.10 | 2.65 | ND | T |
| hsa-miR-182* | 7q32.2 | 1.02 | ND | 3.00 | ND |  |
| hsa-miR-183 | 7q32.2 | 1.45 | 1.09 | 3.35 | 1.08 | T |
| hsa-miR-183* | 7q32.2 | 1.05 | 0.94 | 0.97 | 0.90 |  |
| hsa-miR-96 | 7q32.2 | 1.77 | 1.18 | 4.43 | ND | T |
| hsa-miR-25 | 7q22.1 | 1.14 | 1.09 | 1.13 | 1.08 | T |
| hsa-miR-93 | 7q22.1 | 1.61 | 1.17 | 1.36 | 1.20 | T |
| hsa-miR-196b | 7p15.2 | 1.23 | 1.20 | 1.19 | ND |  |
| hsa-miR-30a | 6q13 | 0.46 | 1.32 | 0.50 | ND | N |
| hsa-miR-30a* | 6q13 | 0.82 | 0.77 | 0.32 | 0.80 | N |
| hsa-miR-30c | 6q13 | 0.67 | 0.67 | 0.75 | 0.65 | N |
| hsa-miR-340* | 5q35.3 | 1.04 | 1.08 | 0.54 | ND |  |
| hsa-miR-143 | 5q32 | 0.42 | 0.30 | 0.69 | 0.47 | N |
| hsa-miR-145 | 5q32 | 0.30 | 0.49 | 0.68 | 0.35 | N |
| hsa-miR-145* | 5q32 | 0.72 | 0.99 | 0.33 | ND |  |
| hsa-miR-378 | 5q32 | 0.49 | 0.40 | 0.40 | 0.67 |  |
| hsa-miR-378* | 5q32 | 0.70 | 1.24 | 0.85 | 0.94 |  |
| hsa-miR-886-3P | 5q31.1 | 1.09 | NP | 1.25 | 1.88 |  |
| hsa-miR-886-5P | 5q31.1 | 1.02 | NP | 1.73 | 1.48 |  |
| hsa-miR-302a | 4q25 | 1.02 | 1.20 | 0.54 | ND |  |
| hsa-miR-218 | 4p15.31 | 0.96 | 1.22 | 0.34 | ND | N |
| hsa-miR-135a* | 3p21.1 | 1.16 | 1.21 | 2.94 | 0.84 |  |
| hsa-miR-375 | 2q35 | 0.40 | 0.70 | 0.55 | 0.57 |  |
| hsa-miR-1258 | 2q31.3 | ND | 0.92 | 2.06 | ND |  |
| hsa-miR-10b | 2q31.1 | 0.68 | 0.91 | 0.49 | 0.90 |  |
| hsa-miR-933 | 2q31.1 | 1.01 | 1.03 | 0.42 | 1.28 |  |
| hsa-miR-29c | 1q32.2 | 0.64 | 0.78 | 0.74 | ND |  |
| hsa-miR-9 | 1q22 | 1.00 | 1.00 | 0.42 | ND | N |
| hsa-miR-552 | 1p34.3 | 1.30 | 1.15 | 2.16 | 1.15 |  |
| hsa-miR-30e | 1p34.2 | 0.68 | 0.79 | 0.65 | ND |  |
| hsa-miR-186 | 1p31.1 | 0.85 | 0.93 | 0.74 | 0.68 |  |
| hsa-miR-372 | 19q13.42 | 0.99 | 1.05 | 1.15 | ND |  |
| hsa-miR-99b* | 19q13.41 | 1.01 | 1.02 | 4.63 | 0.81 |  |
| hsa-miR-638 | 19p13.2 | 0.74 | 0.77 | 1.26 | 0.70 |  |
| hsa-miR-24 | 19p13.13 | 1.44 | 0.99 | 1.01 | 1.32 |  |
| hsa-miR-27a* | 19p13.13 | ND | 0.98 | 4.35 | ND |  |
| hsa-miR-338-3p | 17q25.3 | 0.81 | 1.02 | 0.26 | ND |  |
| hsa-miR-21 | 17q23.1 | 2.47 | 2.11 | 1.11 | 1.42 | T |
| hsa-miR-21* | 17q23.1 | 1.64 | 1.32 | 1.82 | 1.47 |  |
| hsa-miR-195 | 17p13.1 | 0.53 | 0.68 | 0.63 | 0.69 | N |
| hsa-miR-497 | 17p13.1 | 0.61 | ND | 0.35 | 0.81 |  |
| hsa-miR-140-3p | 16q22.1 | 0.73 | 0.82 | 0.72 | 1.04 |  |
| hsa-miR-628-3p | 15q21.3 | ND | 0.69 | 0.62 | 0.95 |  |
| hsa-miR-147b | 15q21.1 | 0.83 | 0.81 | 0.39 | ND |  |
| hsa-miR-1268 | 15q11.2 | 0.85 | 1.01 | 2.58 | 0.81 |  |
| hsa-miR-329 | 14q32.31 | 1.02 | 0.99 | 0.33 | ND |  |
| hsa-miR-376c | 14q32.31 | 0.84 | 0.95 | 0.57 | ND |  |
| hsa-miR-381 | 14q32.31 | 0.90 | 1.08 | 1.08 | 1.14 |  |
| hsa-miR-136 | 14q32.2 | 0.69 | 0.98 | 0.48 | ND |  |
| hsa-miR-139-3p | 11q13.4 | 0.84 | 1.02 | 1.20 | ND |  |
| hsa-miR-139-5p | 11q13.4 | 0.79 | 0.76 | 0.17 | 0.77 | N |
| hsa-miR-675 | 11p15.5 | ND | 0.94 | 2.19 | 0.96 |  |
| hsa-miR-202 | 10q26.3 | 0.79 | 1.05 | 1.12 | ND |  |
| hsa-miR-551b |  | 0.86 | 0.88 | ND | ND |  |
| hsa-miR-649 |  | ND | 1.00 | 2.26 | 0.97 |  |
|  |  |  |  |  |  |  |
| *Refer mainly to systematic review from Ma Y et al (Int J cancer 2012, and references herein) | | | | | | |
| § Coherenty up-modulated in tumor (T) or in normal (N) samples | | | | |  |  |
| NP = not present:; ND = not detected | | |  |  |  |  |
