# Supplementary material for: Comparison of Microarray Platforms for Measuring Differential MicroRNA Expression in Paired Normal/Cancer Colon Tissues
Source: PLoS One. 2012 Sep 13;7(9):e45105. doi: 10.1371/journal.pone.0045105 (PMC3441572; doi:10.1371/journal.pone.0045105)
Supplement: Table S5 — List of the genes co-targeted by at least two miRNAs. (DOCX) [file pone.0045105.s011.docx]

| **Table S5**: List of genes co-targeted by at least two miRNAs | | |  |  |  |  |  |  |  |  |  |  |
| --- | --- | --- | --- | --- | --- | --- | --- | --- | --- | --- | --- | --- |
|  |  |  |  |  |  |  |  |  |  |  |  |  |
|  |  |  |  |  | miR1 |  |  | miR2 |  |  | miR3 |  |
| **Entrez** | **Symbol** | **Name** | **n miRNA** | **name** | **correl** | **q-value** | **name** | **correl** | **q-value** | **name** | **correl** | **q-value** |
| 89958 | C9orf140 | suppressor APC domain containing 2 | 3 | hsa-mir-145 | -0.68158 | 0.031363 | hsa-mir-147b | -0.65462 | 0.042754 | hsa-mir-375 | -0.68639 | 0.029354 |
| 23373 | CRTC1 | CREB regulated transcription coactivator 1 | 3 | hsa-mir-145 | -0.43466 | 0.254783 | hsa-mir-147b | -0.46187 | 0.21919 | hsa-mir-378 | -0.41036 | 0.288722 |
| 54815 | GATAD2A | GATA zinc finger domain containing 2A | 3 | hsa-mir-145 | -0.4383 | 0.251927 | hsa-mir-147b | -0.43194 | 0.257858 | hsa-mir-378 | -0.4742 | 0.202996 |
| 79888 | LPCAT1 | lysophosphatidylcholine acyltransferase 1 | 3 | hsa-mir-145 | -0.5272 | 0.137684 | hsa-mir-375 | -0.68078 | 0.031628 | hsa-mir-378 | -0.58502 | 0.084859 |
| 8566 | PDXK | pyridoxal (pyridoxine, vitamin B6) kinase | 3 | hsa-mir-145 | -0.69682 | 0.025992 | hsa-mir-375 | -0.43694 | 0.253045 | hsa-mir-378 | -0.6013 | 0.073689 |
| 5326 | PLAGL2 | pleiomorphic adenoma gene-like 2 | 3 | hsa-mir-143 | -0.71334 | 0.020369 | hsa-mir-145 | -0.72215 | 0.017781 | hsa-mir-378 | -0.62057 | 0.060742 |
| 5361 | PLXNA1 | plexin A1 | 3 | hsa-mir-147b | -0.48522 | 0.19054 | hsa-mir-375 | -0.70432 | 0.022952 | hsa-mir-378 | -0.53715 | 0.126767 |
| 8140 | SLC7A5 | solute carrier family 7 (amino acid transporter light chain, L system), member 5 | 3 | hsa-mir-145 | -0.56547 | 0.10079 | hsa-mir-147b | -0.74754 | 0.012766 | hsa-mir-375 | -0.80267 | 0.00509 |
| 11033 | ADAP1 | ArfGAP with dual PH domains 1 | 2 | hsa-mir-145 | -0.4846 | 0.19054 | hsa-mir-375 | -0.44304 | 0.245646 |  |  |  |
| 79924 | ADM2 | adrenomedullin 2 | 2 | hsa-mir-375 | -0.50707 | 0.162174 | hsa-mir-378 | -0.4001 | 0.305899 |  |  |  |
| 8659 | ALDH4A1 | aldehyde dehydrogenase 4 family, member A1 | 2 | hsa-mir-145 | -0.44888 | 0.236962 | hsa-mir-378 | -0.74732 | 0.012766 |  |  |  |
| 9949 | AMMECR1 | Alport syndrome, mental retardation, midface hypoplasia and elliptocytosis chromosomal region gene 1 | 2 | hsa-mir-143 | -0.66662 | 0.036691 | hsa-mir-378 | -0.78628 | 0.006012 |  |  |  |
| 23092 | ARHGAP26 | Rho GTPase activating protein 26 | 2 | hsa-mir-143 | -0.43739 | 0.252824 | hsa-mir-145 | -0.40255 | 0.301773 |  |  |  |
| 84913 | ATOH8 | atonal homolog 8 (Drosophila) | 2 | hsa-mir-145 | -0.52913 | 0.135472 | hsa-mir-21* | -0.52453 | 0.140296 |  |  |  |
| 79447 | C16orf53 | chromosome 16 open reading frame 53 | 2 | hsa-mir-145 | -0.42477 | 0.268226 | hsa-mir-378 | -0.63346 | 0.053983 |  |  |  |
| 114904 | C1QTNF6 | C1q and tumor necrosis factor related protein 6 | 2 | hsa-mir-145 | -0.41695 | 0.27887 | hsa-mir-378 | -0.4088 | 0.29139 |  |  |  |
| 1021 | CDK6 | cyclin-dependent kinase 6 | 2 | hsa-mir-145 | -0.59881 | 0.075988 | hsa-mir-378 | -0.58569 | 0.084126 |  |  |  |
| 1951 | CELSR3 | cadherin, EGF LAG seven-pass G-type receptor 3 (flamingo homolog, Drosophila) | 2 | hsa-mir-145 | -0.7525 | 0.011657 | hsa-mir-378 | -0.80663 | 0.004653 |  |  |  |
| 10238 | DCAF7 | DDB1 and CUL4 associated factor 7 | 2 | hsa-mir-143 | -0.5728 | 0.094821 | hsa-mir-145 | -0.58174 | 0.087456 |  |  |  |
| 286148 | DPY19L4 | dpy-19-like 4 (C. elegans) | 2 | hsa-mir-143 | -0.78449 | 0.006079 | hsa-mir-145 | -0.79599 | 0.005451 |  |  |  |
| 2065 | ERBB3 | v-erb-b2 erythroblastic leukemia viral oncogene homolog 3 (avian) | 2 | hsa-mir-143 | -0.48345 | 0.191724 | hsa-mir-145 | -0.53019 | 0.134545 |  |  |  |
| 283991 | FAM100B | family with sequence similarity 100, member B | 2 | hsa-mir-143 | -0.43178 | 0.258065 | hsa-mir-145 | -0.41559 | 0.279526 |  |  |  |
| 139285 | FAM123B | family with sequence similarity 123B | 2 | hsa-mir-145 | -0.41657 | 0.278901 | hsa-mir-378 | -0.40136 | 0.303994 |  |  |  |
| 286077 | FAM83H | family with sequence similarity 83, member H | 2 | hsa-mir-143 | -0.58965 | 0.082234 | hsa-mir-145 | -0.57581 | 0.092263 |  |  |  |
| 157638 | FAM84B | family with sequence similarity 84, member B | 2 | hsa-mir-145 | -0.79929 | 0.005285 | hsa-mir-375 | -0.58759 | 0.083166 |  |  |  |
| 53834 | FGFRL1 | fibroblast growth factor receptor-like 1 | 2 | hsa-mir-147b | -0.67952 | 0.032213 | hsa-mir-375 | -0.50363 | 0.166518 |  |  |  |
| 221937 | FOXK1 | forkhead box K1 | 2 | hsa-mir-375 | -0.52599 | 0.139188 | hsa-mir-378 | -0.46992 | 0.208258 |  |  |  |
| 11226 | GALNT6 | UDP-N-acetyl-alpha-D-galactosamine:polypeptide N-acetylgalactosaminyltransferase 6 (GalNAc-T6) | 2 | hsa-mir-147b | -0.72674 | 0.016968 | hsa-mir-378 | -0.71771 | 0.018931 |  |  |  |
| 160897 | GPR180 | G protein-coupled receptor 180 | 2 | hsa-mir-143 | -0.62422 | 0.058962 | hsa-mir-375 | -0.47649 | 0.201369 |  |  |  |
| 79977 | GRHL2 | grainyhead-like 2 (Drosophila) | 2 | hsa-mir-143 | -0.77358 | 0.007568 | hsa-mir-145 | -0.76385 | 0.009058 |  |  |  |
| 146395 | GSG1L | GSG1-like | 2 | hsa-mir-143 | -0.44049 | 0.248991 | hsa-mir-145 | -0.45041 | 0.235027 |  |  |  |
| 283464 | GXYLT1 | glucoside xylosyltransferase 1 | 2 | hsa-mir-143 | -0.6054 | 0.070682 | hsa-mir-145 | -0.6017 | 0.073492 |  |  |  |
| 3099 | HK2 | hexokinase 2 | 2 | hsa-mir-143 | -0.41689 | 0.27887 | hsa-mir-145 | -0.44095 | 0.248341 |  |  |  |
| 55705 | IPO9 | importin 9 | 2 | hsa-mir-375 | -0.66989 | 0.035444 | hsa-mir-378 | -0.50227 | 0.167664 |  |  |  |
| 23288 | IQCE | IQ motif containing E | 2 | hsa-mir-375 | -0.71721 | 0.019004 | hsa-mir-378 | -0.46935 | 0.208629 |  |  |  |
| 9731 | KIAA0562 | centrosomal protein 104kDa | 2 | hsa-mir-145 | -0.51254 | 0.155154 | hsa-mir-375 | -0.62035 | 0.060798 |  |  |  |
| 57214 | KIAA1199 | KIAA1199 | 2 | hsa-mir-145 | -0.78335 | 0.006079 | hsa-mir-378 | -0.94186 | 2.47E-05 |  |  |  |
| 23367 | LARP1 | La ribonucleoprotein domain family, member 1 | 2 | hsa-mir-143 | -0.52012 | 0.144855 | hsa-mir-145 | -0.55474 | 0.109567 |  |  |  |
| 113251 | LARP4 | La ribonucleoprotein domain family, member 4 | 2 | hsa-mir-143 | -0.56521 | 0.100863 | hsa-mir-145 | -0.56027 | 0.105142 |  |  |  |
| 84823 | LMNB2 | lamin B2 | 2 | hsa-mir-145 | -0.48358 | 0.191629 | hsa-mir-378 | -0.56379 | 0.101967 |  |  |  |
| 9684 | LRRC14 | leucine rich repeat containing 14 | 2 | hsa-mir-145 | -0.65396 | 0.042754 | hsa-mir-378 | -0.43059 | 0.259392 |  |  |  |
| 7867 | MAPKAPK3 | mitogen-activated protein kinase-activated protein kinase 3 | 2 | hsa-mir-143 | -0.64878 | 0.044951 | hsa-mir-145 | -0.68541 | 0.029875 |  |  |  |
| 28985 | MCTS1 | malignant T cell amplified sequence 1 | 2 | hsa-mir-143 | -0.52654 | 0.138536 | hsa-mir-145 | -0.54818 | 0.115152 |  |  |  |
| 4646 | MYO6 | myosin VI | 2 | hsa-mir-143 | -0.68392 | 0.030469 | hsa-mir-145 | -0.78414 | 0.006079 |  |  |  |
| 9683 | N4BP1 | NEDD4 binding protein 1 | 2 | hsa-mir-21* | -0.47228 | 0.205003 | hsa-mir-96 | -0.41994 | 0.276354 |  |  |  |
| 112939 | NACC1 | nucleus accumbens associated 1, BEN and BTB (POZ) domain containing | 2 | hsa-mir-145 | -0.44167 | 0.247289 | hsa-mir-378 | -0.57197 | 0.095685 |  |  |  |
| 81788 | NUAK2 | NUAK family, SNF1-like kinase, 2 | 2 | hsa-mir-143 | -0.48481 | 0.19054 | hsa-mir-145 | -0.46494 | 0.214249 |  |  |  |
| 57460 | PPM1H | protein phosphatase, Mg2+/Mn2+ dependent, 1H | 2 | hsa-mir-145 | -0.85533 | 0.001442 | hsa-mir-378 | -0.90971 | 0.000151 |  |  |  |
| 5581 | PRKCE | protein kinase C, epsilon | 2 | hsa-mir-21 | -0.50164 | 0.168528 | hsa-mir-96 | -0.44529 | 0.242202 |  |  |  |
| 80223 | RAB11FIP1 | RAB11 family interacting protein 1 (class I) | 2 | hsa-mir-143 | -0.54959 | 0.113708 | hsa-mir-145 | -0.52486 | 0.140296 |  |  |  |
| 9609 | RAB36 | RAB36, member RAS oncogene family | 2 | hsa-mir-147b | -0.79639 | 0.005451 | hsa-mir-378 | -0.82172 | 0.003103 |  |  |  |
| 55920 | RCC2 | regulator of chromosome condensation 2 | 2 | hsa-mir-145 | -0.76012 | 0.009892 | hsa-mir-378 | -0.74441 | 0.013534 |  |  |  |
| 11079 | RER1 | RER1 retention in endoplasmic reticulum 1 homolog (S. cerevisiae) | 2 | hsa-mir-143 | -0.77245 | 0.00769 | hsa-mir-375 | -0.41003 | 0.288955 |  |  |  |
| 140730 | RIMS4 | regulating synaptic membrane exocytosis 4 | 2 | hsa-mir-21* | -0.4128 | 0.284082 | hsa-mir-96 | -0.52985 | 0.134977 |  |  |  |
| 57556 | SEMA6A | sema domain, transmembrane domain (TM), and cytoplasmic domain, (semaphorin) 6A | 2 | hsa-mir-21* | -0.51045 | 0.15762 | hsa-mir-96 | -0.70223 | 0.023885 |  |  |  |
| 205564 | SENP5 | SUMO1/sentrin specific peptidase 5 | 2 | hsa-mir-145 | -0.48785 | 0.187 | hsa-mir-378 | -0.62905 | 0.055929 |  |  |  |
| 6513 | SLC2A1 | solute carrier family 2 (facilitated glucose transporter), member 1 | 2 | hsa-mir-143 | -0.5097 | 0.158212 | hsa-mir-378 | -0.55901 | 0.106255 |  |  |  |
| 113235 | SLC46A1 | solute carrier family 46 (folate transporter), member 1 | 2 | hsa-mir-145 | -0.5699 | 0.097271 | hsa-mir-378 | -0.41441 | 0.281462 |  |  |  |
| 6541 | SLC7A1 | solute carrier family 7 (cationic amino acid transporter, y+ system), member 1 | 2 | hsa-mir-145 | -0.51914 | 0.145589 | hsa-mir-378 | -0.6607 | 0.039799 |  |  |  |
| 23657 | SLC7A11 | solute carrier family 7 (anionic amino acid transporter light chain, xc- system), member 11 | 2 | hsa-mir-143 | -0.67242 | 0.034559 | hsa-mir-378 | -0.73954 | 0.014488 |  |  |  |
| 9057 | SLC7A6 | solute carrier family 7 (amino acid transporter light chain, y+L system), member 6 | 2 | hsa-mir-145 | -0.68977 | 0.028237 | hsa-mir-378 | -0.78343 | 0.006079 |  |  |  |
| 6603 | SMARCD2 | SWI/SNF related, matrix associated, actin dependent regulator of chromatin, subfamily d, member 2 | 2 | hsa-mir-143 | -0.68647 | 0.029354 | hsa-mir-145 | -0.69501 | 0.026314 |  |  |  |
| 84530 | SRRM4 | serine/arginine repetitive matrix 4 | 2 | hsa-mir-145 | -0.60022 | 0.074707 | hsa-mir-378 | -0.69348 | 0.026747 |  |  |  |
| 440730 | TRIM67 | tripartite motif containing 67 | 2 | hsa-mir-143 | -0.44221 | 0.246709 | hsa-mir-145 | -0.4334 | 0.255475 |  |  |  |
| 80727 | TTYH3 | tweety homolog 3 (Drosophila) | 2 | hsa-mir-145 | -0.67449 | 0.033886 | hsa-mir-375 | -0.74221 | 0.013859 |  |  |  |
| 64854 | USP46 | ubiquitin specific peptidase 46 | 2 | hsa-mir-143 | -0.45254 | 0.232698 | hsa-mir-145 | -0.45214 | 0.232698 |  |  |  |
| 81839 | VANGL1 | vang-like 1 (van gogh, Drosophila) | 2 | hsa-mir-145 | -0.48083 | 0.19504 | hsa-mir-378 | -0.47992 | 0.195532 |  |  |  |
| 57510 | XPO5 | exportin 5 | 2 | hsa-mir-143 | -0.62668 | 0.057958 | hsa-mir-378 | -0.69943 | 0.024937 |  |  |  |
| 10009 | ZBTB33 | zinc finger and BTB domain containing 33 | 2 | hsa-mir-145 | -0.69985 | 0.024937 | hsa-mir-378 | -0.68508 | 0.029974 |  |  |  |
| 79943 | ZNF696 | zinc finger protein 696 | 2 | hsa-mir-145 | -0.59298 | 0.079736 | hsa-mir-375 | -0.78718 | 0.00593 |  |  |  |
